# Persistent *Mycobacterium tuberculosis* infection in mice requires PerM for successful cell division

**DOI:** 10.7554/eLife.49570

**Published:** 2019-11-21

**Authors:** Ruojun Wang, Kaj Kreutzfeldt, Helene Botella, Julien Vaubourgeix, Dirk Schnappinger, Sabine Ehrt

**Affiliations:** 1 Department of Microbiology and ImmunologyWeill Cornell Medical CollegeNew YorkUnited States; 2 Immunology and Microbial Pathogenesis Graduate ProgramWeill Cornell Graduate School of Medical Sciences, Cornell UniversityNew YorkUnited States; Harvard TH Chan School of Public HealthUnited States; University of the WitwatersrandSouth Africa

**Keywords:** *Mycobacterium tuberculosis*, chronic infection, persistence, cell division, Other

## Abstract

The ability of *Mycobacterium tuberculosis* (Mtb) to persist in its host is central to the pathogenesis of tuberculosis, yet the underlying mechanisms remain incompletely defined. PerM, an integral membrane protein, is required for persistence of Mtb in mice. Here, we show that *perM* deletion caused a cell division defect specifically during the chronic phase of mouse infection, but did not affect Mtb’s cell replication during acute infection. We further demonstrate that PerM is required for cell division in chronically infected mice and in vitro under host-relevant stresses because it is part of the mycobacterial divisome and stabilizes the essential divisome protein FtsB. These data highlight the importance of sustained cell division for Mtb persistence, define condition-specific requirements for cell division and reveal that survival of Mtb during chronic infection depends on a persistence divisome.

## Introduction

The World Health Organization reports that an estimated 23% of the world’s population is latently infected with Mtb (https://www.who.int/tb/publications/global_report/en/). Although asymptomatic and non-contagious, latent infections progress to active disease in 5–15% of cases, causing clinical symptoms and allowing for disease transmission (Pai et al., 2016). The time before active disease develops following exposure to Mtb, ranges from a few months to two years (Behr et al., 2018). And, in regions with minimal TB transmission, Mtb can persist in individuals for decades before causing active disease (Behr et al., 2018). This remarkable ability of Mtb to persist inside its host contributes greatly to its success as a pathogen yet the mechanisms facilitating persistence remain incompletely defined. The term ‘persistence’ used in this report refers to the ability of bacteria to remain viable in the host for a prolonged period of time, and should be distinguished from ‘persister cells’, a subpopulation of transiently antibiotic-tolerant cells (Fisher et al., 2017).

The mouse model has been used to evaluate bacterial factors that allow Mtb to persist within its host. After aerosol infection, Mtb replicates logarithmically in mouse lungs until the onset of adaptive immunity arrests replication, leading to a chronic infection that is contained but not eliminated. Experiments with genetic Mtb mutants identified mycobacterial pathways that are crucial for persistence following the onset of adaptive immunity despite being dispensable for cell replication in the acute phase of infection. Among those pathways, metabolic adaption and modulation of the cell envelope play key roles in Mtb’s persistence (Ehrt et al., 2018; Glickman and Jacobs, 2001).

We have previously reported that PerM, an integral membrane protein with 10 transmembrane helices is essential for Mtb persistence in the mouse model of infection. Disruption of *perM* (*rv0955*) by transposon insertion led to a minor growth defect in the acute phase of mouse infection, but a strong attenuation in the chronic phase (Goodsmith et al., 2015). Further characterization of the mutant by transcriptome analysis, microscopy imaging and drug susceptibility assays suggested that PerM is associated with cell division. However, PerM’s specific roles in cell division and how its absence resulted in a persistence defect in vivo remained unknown.

TB is a complex disease that is highly heterogeneous with regards to outcomes of infection, pathology and nature of bacterial populations (Cadena et al., 2017; Logsdon and Aldridge, 2018; Rego et al., 2017). In fact, the apparent static, non-replicating population that persists in chronically infected mice consists of a mixture of heterogeneous populations of replicating, non-replicating but alive, and dead bacteria (Gill et al., 2009). Using a ‘replication clock’, a plasmid-based reporter of bacterial replication, Gill et al. showed that Mtb replicates during the chronic phase of mouse infection, although at a much slower rate than in the acute phase. This discovery suggests that growth is not only important for increasing the bacterial burden during acute infection, it may also be required for Mtb to persist in chronically infected mice.

Here, we demonstrate that PerM is a mycobacterial divisome component and facilitates cell division by stabilizing FtsB, a conserved divisome protein known to be essential for the initiation of septation (Buddelmeijer et al., 2002; Levin and Losick, 1994; Wu et al., 2018). We document that although PerM is dispensable during the acute phase of mouse infection, it is required for Mtb cell division during chronic mouse infection and in host-relevant stress conditions, during which maintaining an optimal FtsB level becomes crucial. To our knowledge, PerM represents the first reported bacterial divisome component that is conditionally essential for cell division at different stages of mouse infection.

## Results

### Mtb requires PerM for optimal cell replication during chronic mouse infection

We previously reported that an Mtb *perM* transposon mutant failed to persist in the chronic phase of mouse infection (Goodsmith et al., 2015). To distinguish whether this was the result of slowed bacterial replication or enhanced killing by the host, we constructed a *perM* deletion strain (*ΔperM*) (Xu et al., 2017) and determined cell replication rates of WT and the *ΔperM* mutant using the ‘replication clock’ plasmid (Gill et al., 2009). Measurements of growth and plasmid loss showed that the two strains replicated at a similar rate in standard in vitro growth conditions (Figure 1—figure supplement 1).

**Figure 1.**
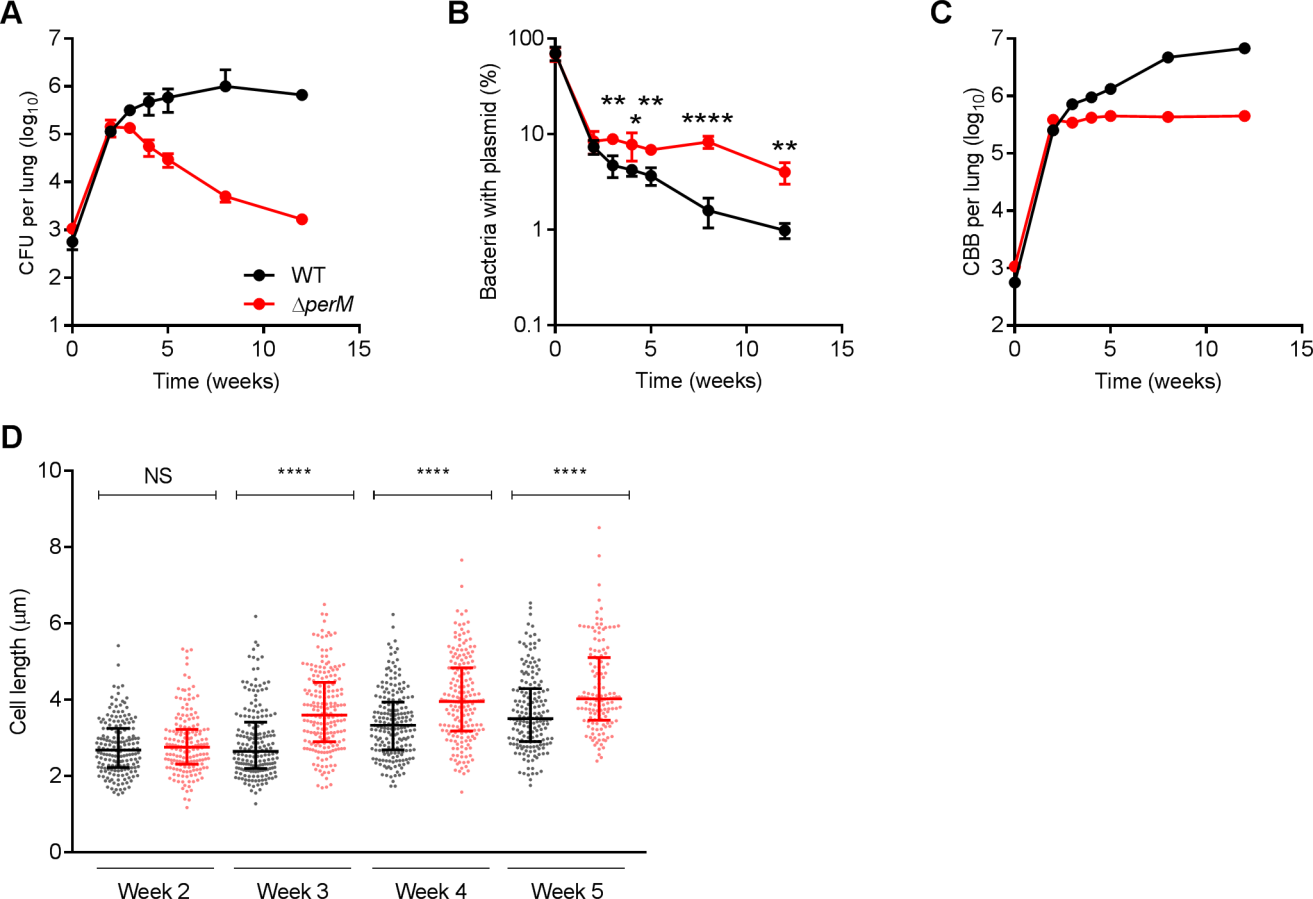
Mtb requires PerM for optimal cell division during chronic mouse infection. (**A**) Bacterial titers in lungs of C57BL/6 mice infected with WT Mtb or the *∆perM* mutant containing the replication clock plasmid pBP10 at the indicated time points post-infection. Data are means ± SD of five mice. (**B**) Fractions of bacteria containing the pBP10 plasmid at the indicated time points. Data are means ± SD of five mice. P-values were calculated using multiple *t* tests and adjusted for multiple comparisons. *, adj-P <0.05; **, adj-P <0.01; ****, adj-P <0.0001. (**C**) Calculated cumulative bacterial burden (CBB) in mouse lungs. (**D**) Scatter plots of cell length of WT Mtb (black) or the *∆perM* mutant (red) in acid-fast stained lung sections at the indicated time points. The middle lines represent the medians and the top and bottom lines represent the 25^th^ and 75^th^ percentiles. P-values were calculated using Kruskal-Wallis test and corrected for multiple comparisons. ****, adj-P <0.0001. Data in (**A–C**) are from one experiment, and data in (**D**) are representative of two independent experiments. Figure 1—source data 1.Summary statistics of Figure 1D.

During the initial two weeks after aerosol infection with approximately 1000 CFU, WT and *ΔperM* grew logarithmically in mouse lungs (Figure 1A). As expected, this was accompanied by a decrease in the frequency of bacteria carrying the ‘replication clock’ plasmid (Figure 1B). The rate of plasmid loss was similar between WT and *ΔperM* (Figure 1B), resulting in a calculated doubling time of around 20 hr for both strains (Figure 1—figure supplement 2A). During the first two weeks of infection, cell replication rates exceeded death rates in both Mtb strains and led to an increase in bacterial burden (Figure 1—figure supplement 2B).

**Figure 1—figure supplement 1.**
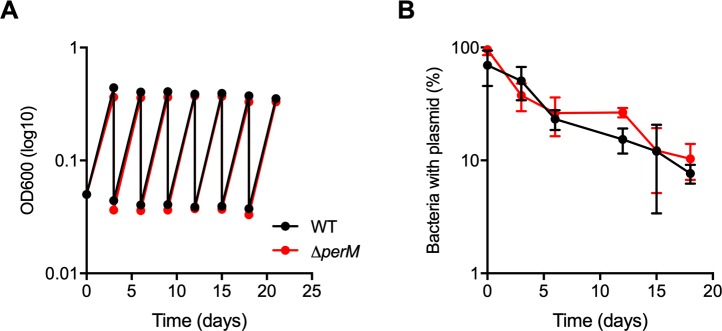
Mtb WT and *∆perM* replicate similarly in vitro. (**A**) Growth measured by optical density of serially diluted WT-pBP10 and *∆perM*-pBP10 cultures. Cultures were diluted every three days to allow for continuous culturing. Data are means ± SD of triplicates (error bars are too small to be detected).(**B**) Plasmid loss from Mtb WT and the *∆perM *mutant in log phase continuous cultures in the absence of antibiotic selection. Data are means ± SEM of triplicates. Data in (**A and B**) are representative of two independent experiments.

**Figure 1—figure supplement 2.**
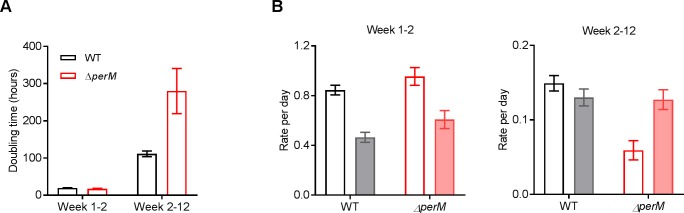
Mtb *∆perM* replicates more slowly than WT in the chronic phase of mouse infection. (**A**) Calculated doubling time of WT-pBP10 and *∆perM*-pBP10 recovered from mouse lungs during the indicated time intervals. Data are represented as doubling time ± SE. (**B**) Calculated bacterial replication (empty bars) and death (filled bars) rates of WT-pBP10 and *∆perM*-pBP10 recovered from mouse lungs during the indicated time intervals. Calculations of the cell replication and death rates are described in Materials and methods. Data are replication or death rates ± SE. Statistical significance for cell replication rates (**r**) was computed using analysis of covariance (ANCOVA). *P* = 0.1995 comparing r of WT and *∆perM* during week 1-2. *P* < 0.0001 comparing r of WT and *∆perM* during week 2-12. Data in (**A and B**) are from one experiment.

**Figure 1—figure supplement 3.**
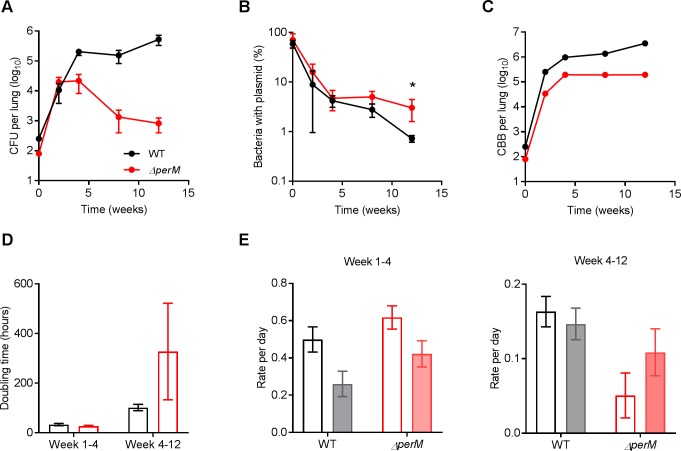
Replication clock experiment with low Mtb inoculum reveals slower cell replication of *∆perM* than WT in the chronic phase of mouse infection. (**A**) Bacterial burden in lungs of C57BL/6 mice infected with WT-pBP10 or *∆perM*-pBP10. Data are means ± SD of five mice. (**B**) Fractions of bacteria containing the pBP10 plasmid at the indicated time points. Data are means ± SD of five mice. P-values were calculated using multiple *t* tests and adjusted for multiple comparisons. *, adj-P <0.05. At week 8, adj-P equals 0.066. (**C**) Calculated cumulative bacterial burden (CBB) in mouse lungs. (**D**) Calculated doubling time during the indicated time intervals. Data are doubling time ± SE. (**E**) Calculated cell replication (empty bars) and death (filled bars) rates during the indicated time intervals. Data are rates ± SE. Statistical significance for cell replication rates (**r**) was computed using ANCOVA. *P* = 0.2102 comparing r of WT and *∆perM* during week 1-4. *P* = 0.0047 comparing r of WT and *∆perM* during week 4-12. Data in (**A–E**) are from one experiment.

From 2 to 12 weeks post infection, WT Mtb persisted with stable bacterial titers, while *ΔperM* titers decreased (Figure 1A). Both strains lost the ‘replication clock’ plasmid more slowly than during the acute phase of infection, but plasmid loss was significantly slower in *ΔperM* than in WT (Figure 1B). The calculated doubling time of *ΔperM* was 280 ± 61 hr, more than twice the doubling time of WT, which was 111 ± 8 hr (Figure 1—figure supplement 2A). Consistently, the cell replication rate of *ΔperM* was significantly lower than that of WT while death rates were comparable (Figure 1—figure supplement 2B). Next, we quantified the cumulative bacterial burden (CBB), defined as the total number of bacteria in the mouse lung including live, dead and those cleared by host immunity (Gill et al., 2009). We found that the CBB of WT increased steadily during weeks 2–12. In marked contrast, the CBB of *ΔperM* increased only slightly during the same time interval (Figure 1C). A second mouse infection with approximately 100 CFU administered by aerosol yielded similar results (Figure 1—figure supplement 3). Overall, these data demonstrate that the failure of *ΔperM* to persist in the chronic phase of mouse infection can be attributed to a cell replication defect.

### PerM facilitates cell division in Mtb during chronic mouse infection

We quantified bacterial cell length in acid fast-stained infected lung sections. At week two, WT and *ΔperM* had comparable median cell lengths (Figure 1D). At week three, *ΔperM* cells were significantly longer than WT cells and this difference in cell length remained at weeks four and five. These measurements strongly suggest that the observed cell replication defect is associated with impaired cell division during the chronic phase of mouse infection.

### Depletion of PerM leads to defects in septum formation and resolution in *M. smegmatis*

**Figure 2.**
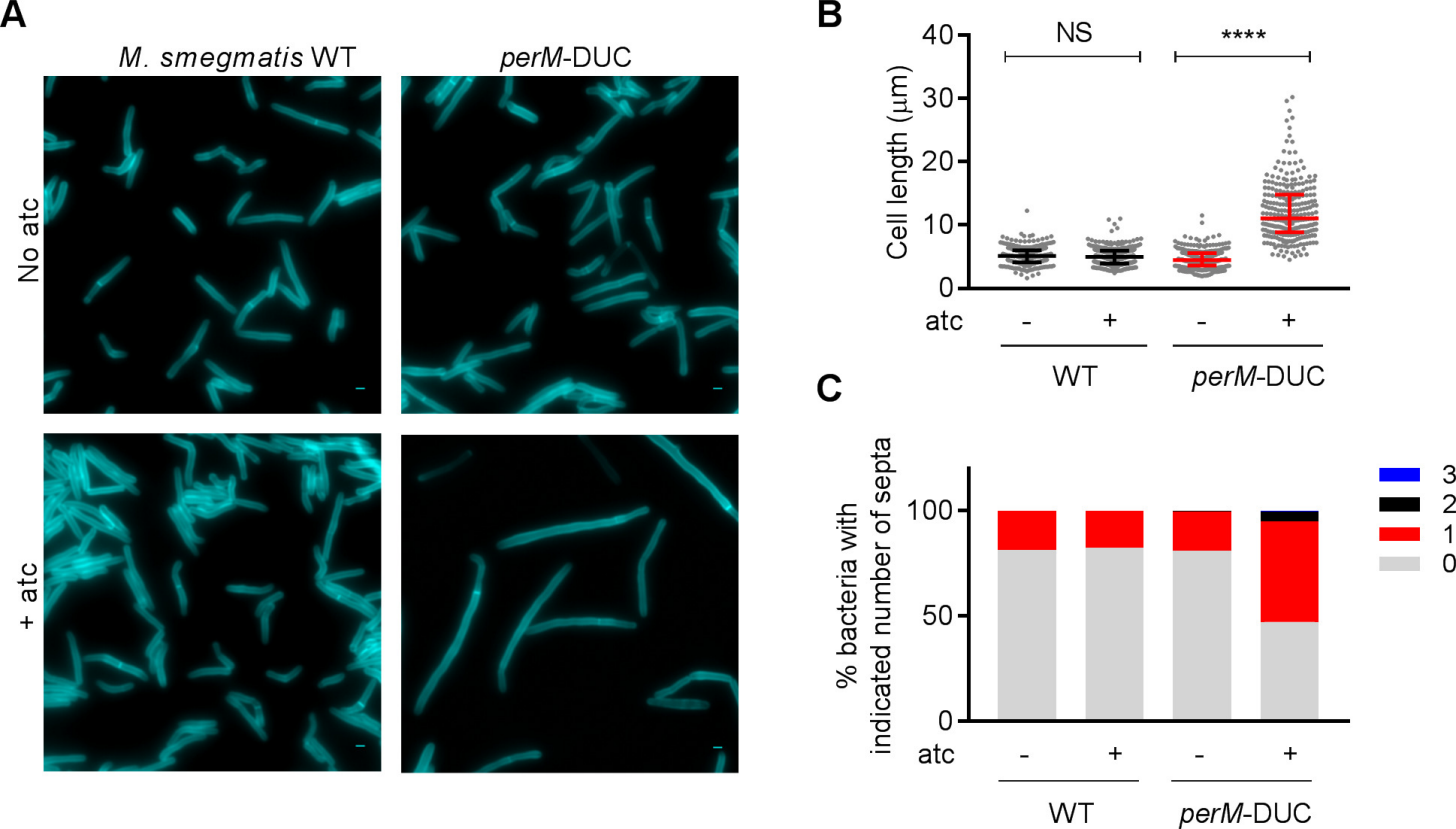
Depletion of PerM leads to a septation defect in *M. smegmatis.* (**A**) Representative microscopy images of WT *M. smegmatis* and the *perM*-DUC mutant after 9 hr of incubation in 7H9 medium supplemented with 1 mM HADA and in the absence or presence of 200 ng/ml atc. Scale bar, 1 µm. (**B**) Scatter plots of bacterial cell length of *M. smegmatis* strains from (**A**). The middle lines represent the medians and the top and bottom lines represent the 25^th^ and 75^th^ percentiles. P-values were computed using Kruskal-Wallis test and adjusted for multiple comparisons. ****, adj-P <0.0001. (**C**) Quantification of bacterial cells that contain 0, 1, 2 or 3 septa of *M. smegmatis* strains from (**A**). Data in (**A–C**) are representative of two independent experiments. Figure 2—source data 1.Summary statistics of Figure 2B.

To explore how PerM facilitates cell division, we studied its homolog in *M. smegmatis*, a non-pathogenic mycobacterium; MSMEG_5517 (PerM_msm_) shares 73% identity with PerM from Mtb. Unexpectedly, we failed to isolate a *perM_msm_* deletion mutant, which suggested that *perM_msm_* is essential for growth of *M. smegmatis*. We therefore constructed a PerM dual-control (DUC) mutant (Figure 2—figure supplement 1A–C), in which PerM expression is controlled by both transcriptional silencing and proteolytic degradation in response to anhydrotetracycline (atc) (Kim et al., 2013; Schnappinger et al., 2015). The number of viable cells remained constant in the PerM-depleted population, indicating that PerM depletion was bacteriostatic over the course of the experiment (Figure 2—figure supplement 1D). PerM depletion resulted in an increase of median cell length from 4.46 µm to 11.1 µm in *M. smegmatis* (Figure 2A,B). Additionally, peptidoglycan labeling with the fluorescent D-alanine analog HCC‐amino‐D‐alanine (HADA) revealed that the elongated cells formed filaments, with the majority containing either none or only one septum (Figure 2A,C) (Kuru et al., 2012; Kuru et al., 2015). DNA staining with SYTO 13 revealed separated nucleoids spanning the entire length of each bacterium, suggesting that PerM depletion did not affect DNA segregation (Figure 2—figure supplement 2). The phenotypes associated with PerM depletion in *M. smegmatis* demonstrate that PerM is involved in septation.

**Figure 2—figure supplement 1.**
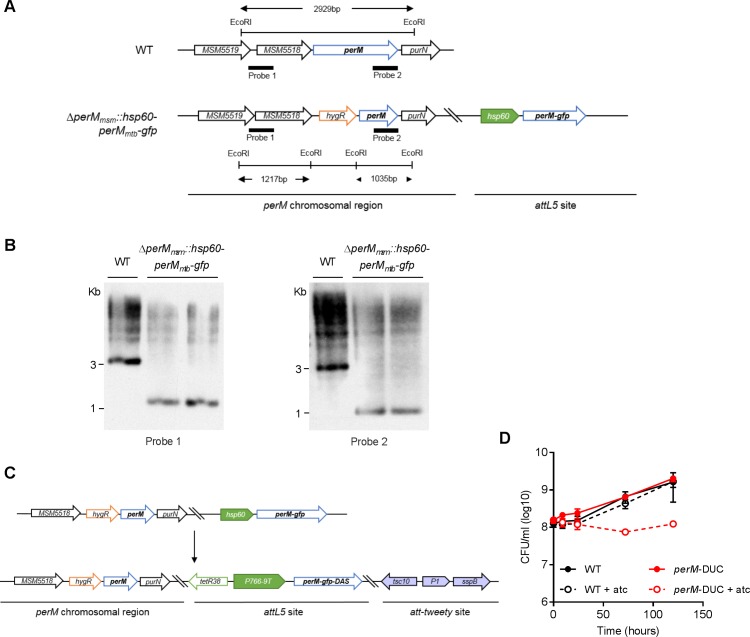
Construction and characterization of a PerM_msm_ depletion mutant (*perM*-DUC). (**A**) Map of *perM_msm_* genomic region in WT and the att-site mutant *∆perM_msm_::hsp60-perM_mtb_-gfp*. To construct the att-site mutant, we first generated a merodiploid strain by integrating a copy of *perM_mtb_-gfp* into the attL5 site expressed under the constitutive hsp60 promoter. Next, we replaced the first 971 bp of *perM_msm_* with a hygromycin resistance cassette using homologous recombination. (**B**) Southern blots of genomic DNA from WT and two att-site mutant candidates after digestion with *Eco*RI and probed with probe 1 (left) and probe 2 (right) as marked in (**A**). Bands at around 1 kb from the two candidates showed the successful deletion of *perM_msm_*. (**C**) Schematic of constructing the *perM*-DUC mutant from the *perM* att-site mutant. We first replaced the construct in the attL5 site of *∆perM_msm_::hsp60-perM_mtb_-gfp* with DAS tagged *perM-gfp* expressed under the P766-9T promoter and controlled by reverse tet repressor. A construct that expresses the SspB adaptor protein was integrated into the att-tweety site. (**D**) Bacterial titers of WT and *perM*-DUC cultured in standing 7H9 medium with and without atc. At the indicated time points, samples were collected and CFU recovered on 7H10 agar that contains 0.4% charcoal to remove carryover of atc. Data are means ± SD of three replicates and representative of two independent experiments.

**Figure 2—figure supplement 2.**
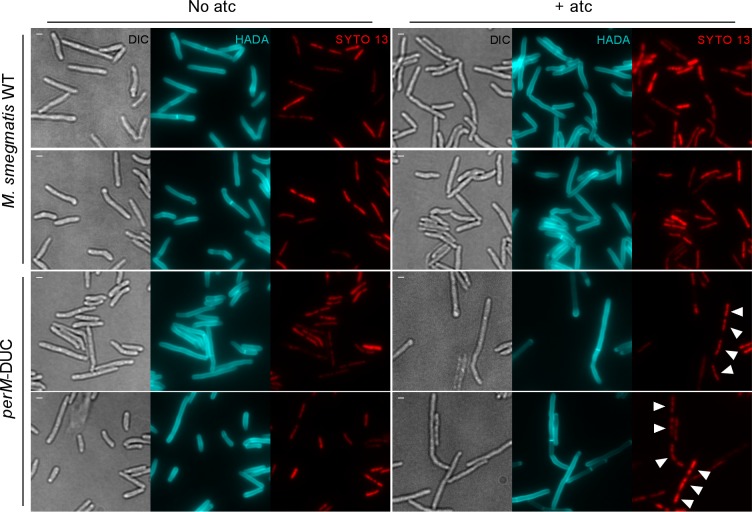
Nucleoid morphology of WT *M. smegmatis* and *perM*-DUC in the absence and presence of atc. Representative microscopy images of WT and *perM*-DUC incubated in 7H9 medium containing 1 mM HADA and in the absence or presence of 200 ng/ml atc for 9 hr. Cells were stained with 5 µM SYTO 13 to label the DNA. Arrowheads point to multiple stained nucleoids (visualized as distinct globular structures) within the *perM*-DUC mutant incubated in the presence of atc. Scale bar, 1 µm.

**Figure 3.**
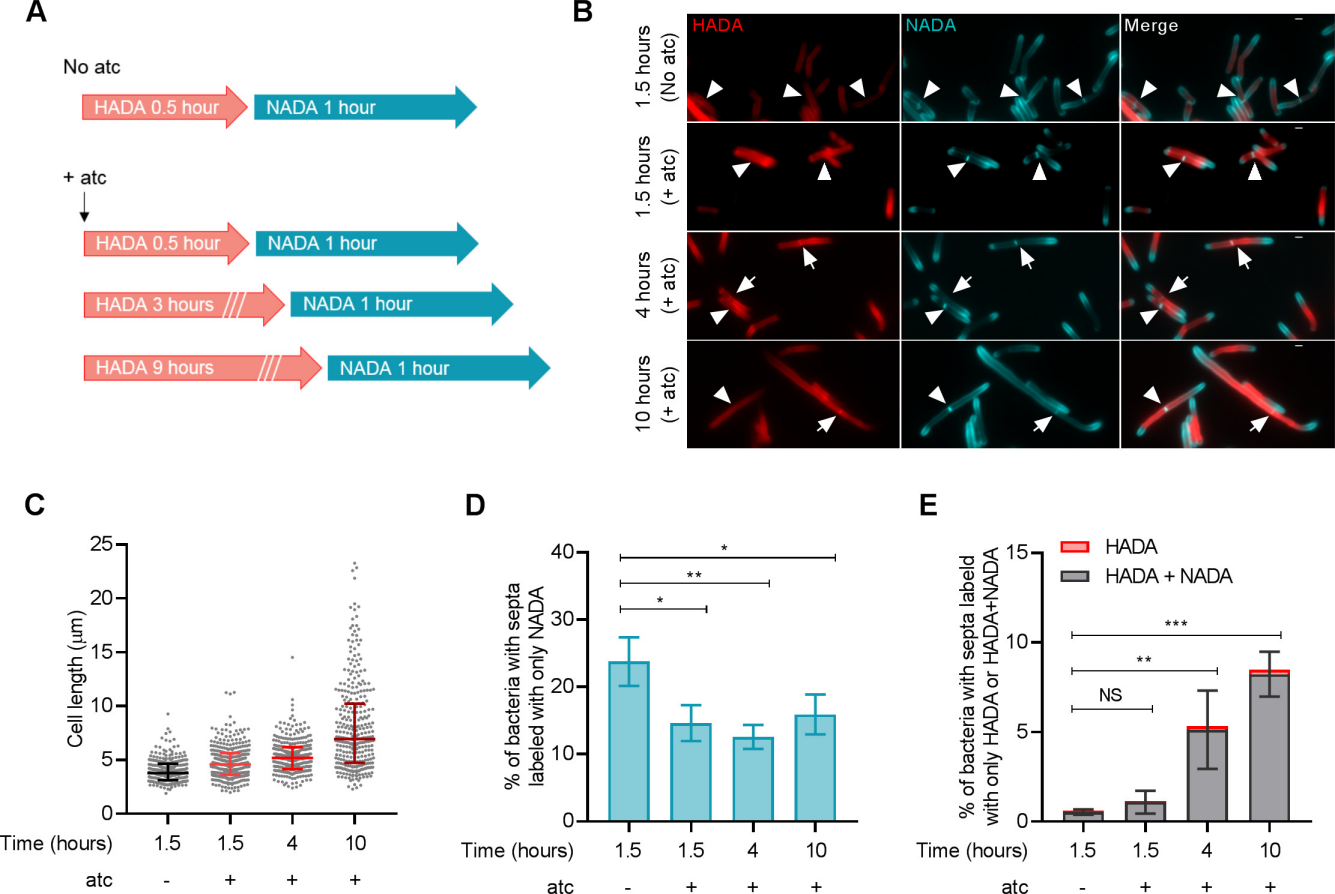
PerM depletion leads to defects in septum formation and resolution in *M. smegmatis*. (**A**) Schematic of peptidoglycan labeling of *M. smegmatis perM*-DUC. Bacterial cells were incubated in growth medium containing 1 mM HADA and 200 ng/ml atc for 0.5, 3, or 9 hr. Cells were then washed to remove excess HADA and incubated with 1 mM NADA and 200 ng/ml atc for 1 hr. *PerM*-DUC cells incubated in growth medium containing D-alanine probes but without atc were used as controls. (**B**) Representative microscopy images of *perM*-DUC after the sequential peptidoglycan labeling. Arrowheads point to septa labeled with NADA only; and arrows point to septa co-labeled with HADA and NADA. Scale bar, 1 µm. (**C**) Scatter plots of *perM*-DUC cell lengths from (**B**). Lines represent median and the 25^th^ and 75^th^ percentiles. Data are representative of three biological replicates. (**D**) Proportion of bacteria that contain septa labeled exclusively with NADA. Lines indicate means ± SD of three independent experiments. (**E**) Proportion of bacteria that contain septa labeled exclusively with HADA or co-labeled with NADA. Lines indicate means ± SD of septa labeled with HADA+NADA from three independent experiments. Statistics are reported on the HADA+NADA datasets. For data in (**D and E**), P-values were computed using ANOVA and adjusted for multiple comparisons. *, adj-P <0.05; **, adj-P <0.01; ***, adj-P <0.001. Figure 3—source data 1.Summary statistics of Figure 3C. Figure 3—source data 2.Summary of Figure 3D and 3E.

We then monitored septum formation and resolution in *M. smegmatis* by sequential peptidoglycan labeling with HADA and a second fluorescent D-alanine analog, NBD‐amino‐D‐alanine (NADA) (Figure 3A). As expected, incubation of *perM*-DUC with atc led to increased cell length (Figure 3B,C); the median cell length increased from 3.79 μm (no atc) to 4.598 μm, 5.191 μm and 6.948 μm after 1.5, 4 and 10 hr of atc treatment. Next, we quantified the formation of new septa (labeled with NADA alone) and the resolution of old septa (labeled with HADA alone or both D-alanine probes) after PerM depletion. The percentage of septa labeled with NADA alone declined from 23.77% (no atc) to 14.62%, 12.56% and 15.92% after 1.5, 4 and 10 hr incubation with atc (Figure 3D), suggesting that PerM-depleted cells had an impaired ability to form new septa. In contrast, the proportion of HADA-containing septa increased with time, from 0.61% (no atc) to 1.14%, 5.34% and 8.48% after 1.5, 4 and 10 hr atc treatment (Figure 3E). Collectively, these results indicate that PerM depletion affects both septum formation and cell separation in *M. smegmatis*, causing the bacteria to form filaments.

### Ectopic FtsB expression rescues the phenotypes caused by PerM depletion in *M. smegmatis* and PerM deletion in Mtb

Septation requires the coordinated activities of cell wall biosynthetic and lytic enzymes (Kieser and Rubin, 2014). To identify proteins that might rescue the septation defects caused by PerM depletion in *M. smegmatis*, we performed a forward genetic screen. We transformed *perM*-DUC with an Mtb genomic library and selected for colonies able to grow on agar containing atc, a non-growth-permissive condition for the *perM*-DUC mutant. The plasmids that rescued growth of *perM*-DUC contained either *rv0955* (*perM*) or *rv1024*.

*Rv1024* encodes the Mtb homolog of FtsB (FtsB_mtb_), an integral membrane protein of the bacterial divisome that is conserved across different bacterial species (Buddelmeijer et al., 2002; Levin and Losick, 1994; Wu et al., 2018). In model bacteria such as *E. coli*, FtsB is recruited to the septum as part of a complex that also contains FtsQ and FtsL, two other bitopic proteins essential for cell division (Buddelmeijer and Beckwith, 2004; Glas et al., 2015). The FtsQLB complex is thought to drive septation by stimulating peptidoglycan synthesis by the peptidoglycan synthases FtsI and FtsW along with their associated proteins (Kureisaite-Ciziene et al., 2018; Liu et al., 2015; Taguchi et al., 2019; Tsang and Bernhardt, 2015). *M. smegmatis* encodes homologs of all three proteins. Like in *E. coli*, depletion of FtsQ, FtsL or FtsB in *M. smegmatis* resulted in morphological changes indicative of impaired septation (Buddelmeijer et al., 2002; Carson et al., 1991; Guzman et al., 1992; Wu et al., 2018).

**Figure 4.**
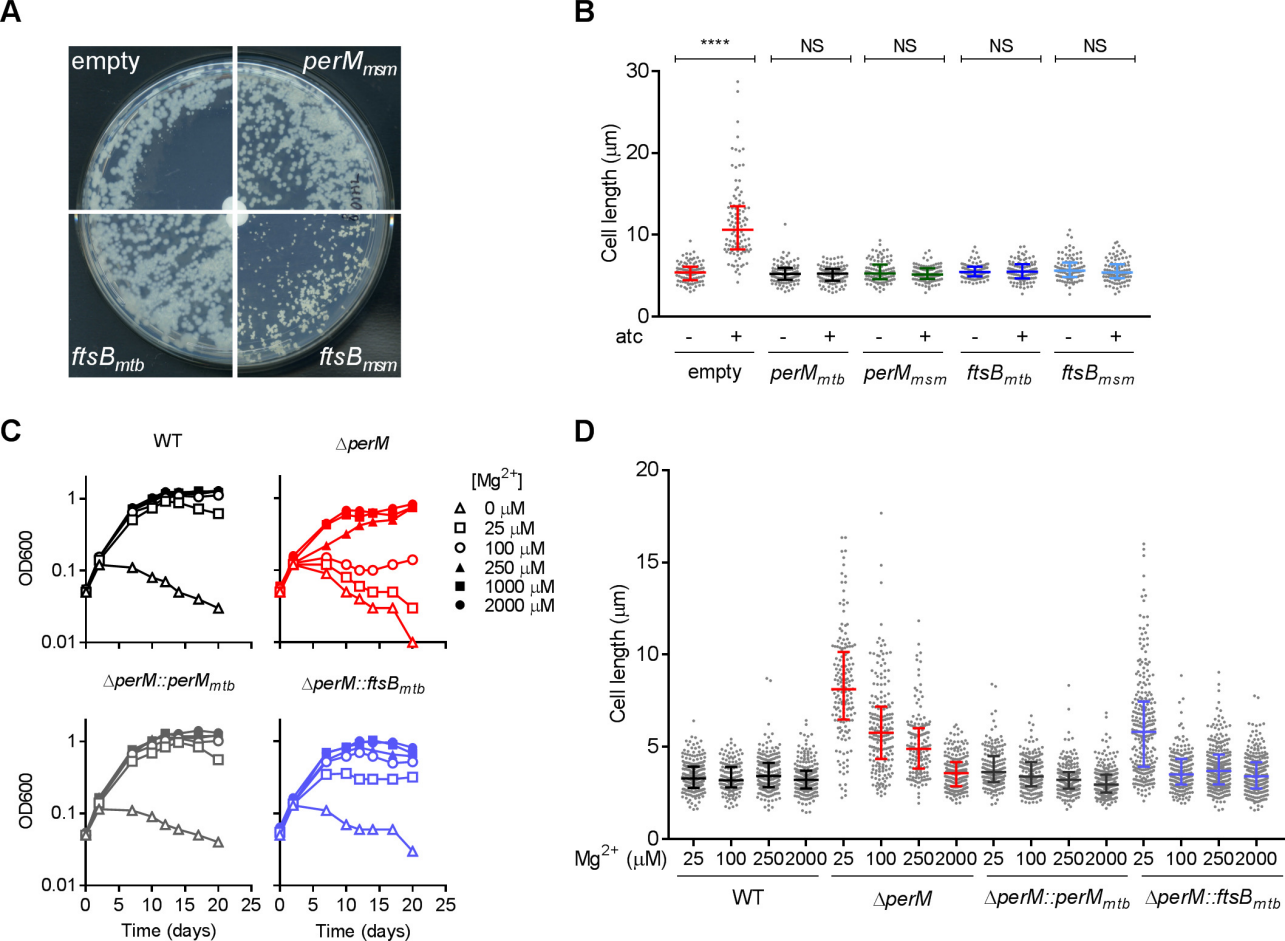
Ectopic *ftsB* expression rescues the phenotypes caused by PerM depletion in *M. smegmatis* and *perM* deletion in Mtb. (**A**) *M. smegmatis perM-*DUC transformed with a control plasmid or plasmids that express *perM_msm_*, *ftsB_mtb_* or *ftsB_msm_* under the constitutive hsp60 promoter were cultured on 7H10 agar. The paper disks in the center of the plates contained 50 ng atc, which diffused and created an atc gradient from the center to the periphery. Images of plates were taken on day 4 (*perM*-DUC-control, *perM-*DUC*-perM_msm_* and *perM-*DUC*-ftsB_mtb_*) or day 7 (*perM-*DUC*-ftsB_msm_*). (**B**) Scatter plots of the lengths of *perM*-DUC-control, *perM-*DUC*-perM_mtb_, perM-*DUC*-perM_msm_*, *perM-*DUC*-ftsB_mtb_* and *perM-*DUC*-ftsB_msm_* incubated in 7H9 medium without and with atc for 24 hr. The lines represent the 25^th^, 50^th^ and 75^th^ percentiles. P-values were computed using Kruskal-Wallis test and adjusted for multiple comparisons. ****, adj-P <0.0001. (**C**) Growth curves of Mtb incubated in chelated Sauton’s medium supplemented with Mg^2+^ at the indicated concentrations. (**D**) Scatter plots of Mtb cell lengths after 5 days of incubation in Sauton’s medium containing Mg^2+^ at the indicated concentrations. The lines represent the 25^th^, 50^th^ and 75^th^ percentiles. P-values were computed using Kruskal-Wallis test and corrected for multiple comparisons. The adjusted P-values are <0.0001 for the following comparisons: WT with *∆perM* at 25 µM, 100 µM, 250 µM Mg^2+^, WT with *∆perM::ftsB_mtb_* at 25 µM Mg^2+^; *∆perM* with *∆perM::ftsB_mtb_* at 25 µM Mg^2+^. Adj-P = 0.0762, 0.1095 and 0.7284 comparing WT to *∆perM::ftsB_mtb_* at 100 µM, 250 µM and 2000 µM Mg^2+^, respectively. Data in (**A–C**) are representative of two independent experiments, and data in (**D**) are from one experiment. Figure 4—source data 1.Summary statistics of Figure 4B. Figure 4—source data 2.Summary statistics of Figure 4D.

FtsB_mtb_ is 72% identical to FtsB_msm_, and ectopic expression of either protein from a multicopy plasmid rescued the growth defect caused by PerM depletion in *M. smegmatis* (Figure 4A). Furthermore, ectopically-expressed FtsB_mtb_ or FtsB_msm_ reversed the cell elongation phenotype as did complementation with *perM_mtb_* or *perM_msm_* (Figure 4B). Therefore, PerM likely acts on FtsB to facilitate septum formation in *M. smegmatis*. Moreover, FtsB_mtb_ and FtsB_msm_ appear to be functionally conserved.

As we reported previously (Goodsmith et al., 2015), PerM is required for cell replication and survival of Mtb in media with reduced magnesium (Mg^2+^) and Mtb lacking PerM elongated in response to Mg^2+^ limitation (Figure 4C,D). Both the growth and elongation phenotypes were, to a large extent, rescued by ectopic expression of FtsB, indicating that PerM likely functions via FtsB in Mtb as well.

### PerM interacts with FtsB

To assess the molecular mechanism by which PerM contributes to cell division and to determine if FtsB and PerM interact physically, we immunoprecipitated FtsB_mtb_ containing an N-terminal Flag-tag from Mtb protein lysates and identified its interactors using mass spectrometry (Table 1). PerM_mtb_ co-immunoprecipitated with FtsB_mtb_, suggesting that the two proteins are part of the same complex. This interaction was verified in *M. smegmatis* strains that co-expressed PerM_mtb_ and FtsB_mtb_ with different protein tags (Figure 5—figure supplement 1). The mass spectrometry data also revealed that FtsB interacts with FtsQ in Mtb (Table 1). Similar to its homologs in *E. coli* and *B. subtilis*, FtsQ_mtb_ has been shown to be a septal-localizing integral membrane protein that modulates cell division (Buddelmeijer and Beckwith, 2004; Jain et al., 2018; Katis et al., 2000). FtsB, FtsL and FtsQ form a complex in *E. coli* (Buddelmeijer and Beckwith, 2004; Kureisaite-Ciziene et al., 2018), and here we provide evidence that the interaction between FtsB and FtsQ is conserved in mycobacteria.

**Table 1.** Mass spectrometry identification of protein interaction partners of FtsB in Mtb. Table 1—source data 1.Mass spectrometry data of FtsB pulldown in Mtb.

Rv #	Gene	Annotation	Sum total spectrum count
Rv1687c		ABC transporter ATP-binding protein	20
Rv2901c		Hypothetical protein	16
Rv1280c	*oppA*	Probable periplasmic oligopeptide-binding lipoprotein OppA	16
Rv2151c	*ftsQ*	Cell division protein FtsQ	15
Rv1697		Hypothetical protein	14
Rv3330	*dacB1*	Probable penicillin-binding protein DacB1	14
Rv1698	*mctB*	Outer membrane protein MctB	14
Rv1266c	*pknH*	Serine/threonine protein kinase	12
Rv2748c	*ftsK*	Cell division protein FtsK	11
Rv0955	*perM*		11
Rv0050	*ponA1*	Peptidoglycan glycosyltransferase	10
Rv3493c		MCE-associated alanine and valine rich protein	10
Rv0497		Probable conserved transmembrane protein	10
Rv3212		Conserved alanine valine rich protein	9
Rv0046c	*ino1*	Inositol-1-phosphate synthase	8

Flag-FtsB_mtb_ was immunoprecipitated from whole-cell lysates of Mtb. Whole-cell lysates of an Mtb strain constitutively expressing only the Flag-tag served as control. The protein interaction partners were identified by mass spectrometry and data are from two independent biological replicates.

### FtsB localizes to the mid-cell independently of PerM

**Figure 5.**
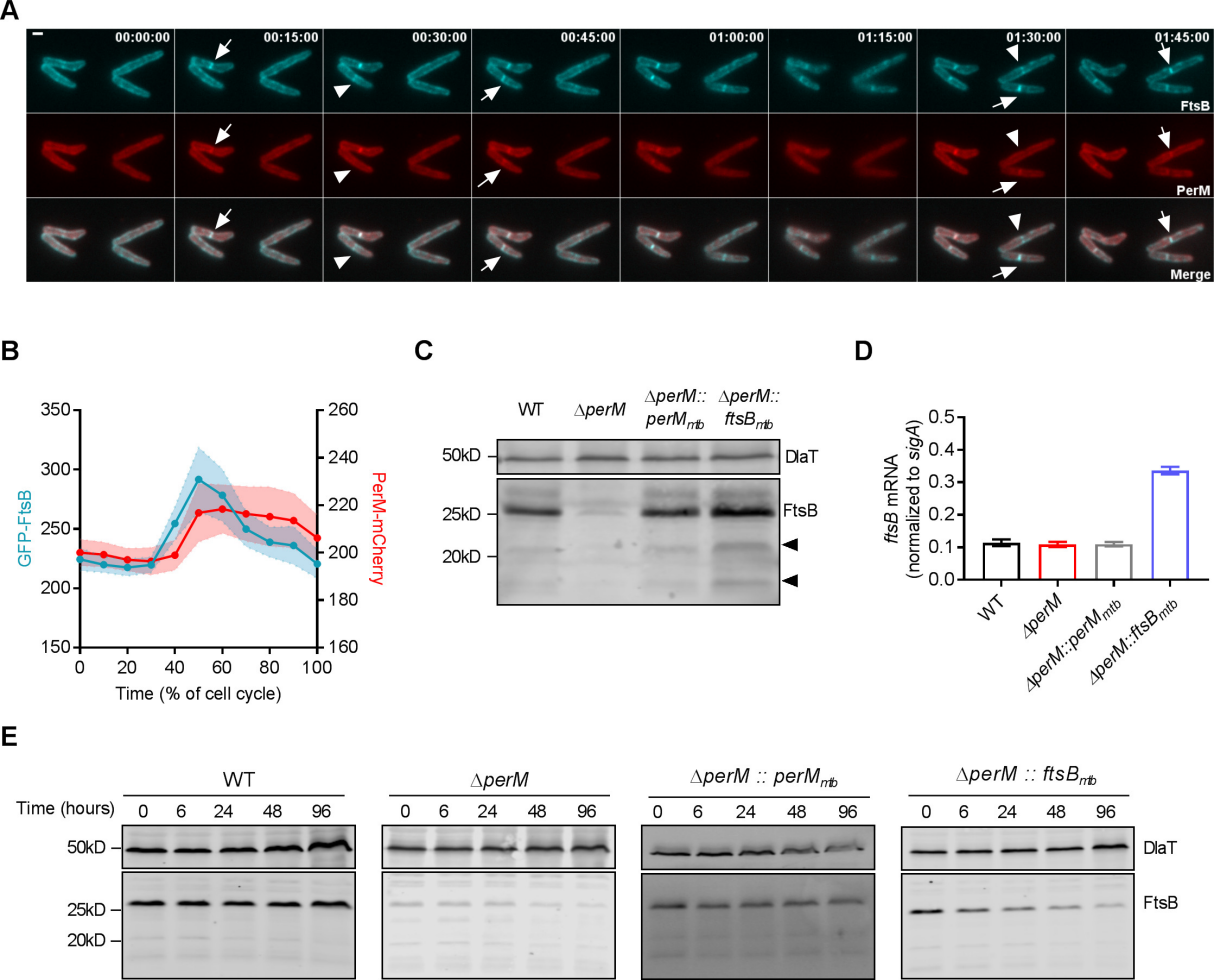
PerM co-localizes with FtsB and stabilizes FtsB. (**A**) Representative image series from time lapse movies of replicating *M. smegmatis* constitutively expressing both GFP-FtsB_mtb_ and PerM_mtb_-mCherry over the duration of 1 hr and 45 mins. Arrows point to the presence of both FtsB and PerM at the mid-cell, and arrowheads point to the presence of FtsB and lack of PerM at the mid-cells. Scale bar, 1 µm. Numbers in the upper right corner indicate time. (**B**) Measurements of the maximum fluorescence intensities of GFP-FtsB and PerM-mCherry as a function of the cell cycle from time lapse movies shown in (**A**). The lines and shaded areas indicate means and the 95% confidence intervals of 20 bacteria. (**C**) FtsB protein in whole-cell lysates of log-phase Mtb measured by western blotting with anti-FtsB antibody. Arrowheads point to cleavage products of FtsB. Dihydrolipoamide acyltransferase (DlaT) was used as loading control. (**D**) *FtsB* mRNA in log-phase Mtb culture measured by quantitative real time PCR. mRNA levels were normalized to expression of the housekeeping gene *sigA*. Data are means ± SD of triplicates. (**E**) Detection of FtsB in whole-cell lysates collected from Mtb strains by western blotting with anti-FtsB antibody. 20 µg/ml chloramphenicol were added to each culture at time 0 to inhibit protein synthesis and samples were collected at the indicated time points. Western blotting with anti-DlaT was used as loading control. Data in (**C–E**) are representative of two independent experiments. Biological replicates of Figure 5C and E are shown in Figure 5—figure supplement 3.

We next investigated the spatiotemporal dynamics of FtsB and PerM during the cell cycle by co-expressing FtsB_mtb_ and PerM_mtb_ fused to different fluorescent proteins in *M. smegmatis*. Time-lapse microscopy showed that FtsB localized to the mid-cell ahead of PerM (Figure 5A). PerM localized to the same site approximately 15 min later with both proteins present at the mid-cell prior to septum constriction. We quantified the fluorescence intensities at the mid-cell and observed that the FtsB signals started to increase at ~30% and peaked at ~50% of the cell cycle, whereas the PerM signals increased at ~40% and stabilized by ~50% of the cell cycle (Figure 5B), confirming that FtsB precedes PerM at the division site. This sequential localization was not an artifact caused by the fluorescent tags, as exchanging the tags resulted in the same order, with FtsB detectable at the mid-cell before PerM (Figure 5—figure supplement 2A). In addition, in *M. smegmatis* lacking PerM but ectopically expressing a second copy of FtsB, FtsB also localized to the septum (Figure 5—figure supplement 2B). This suggests that the localization of FtsB to mid-cell does not depend on the presence of PerM.

**Figure 5—figure supplement 1.**
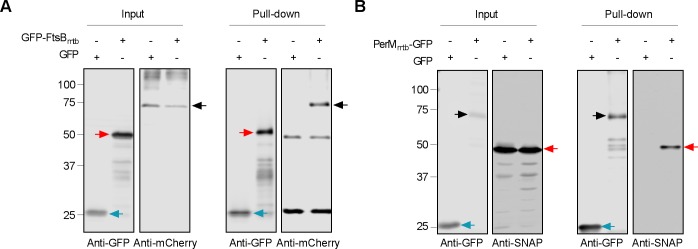
FtsB_mtb_ and PerM_mtb_ interact in vivo. (**A**) Co-immunoprecipitation of FtsB_mtb_ and PerM_mtb_ from whole-cell protein lysates of *M. smegmatis ∆perM::perM_mtb_-mCherry::gfp-ftsB_mtb_*. Lysates from *M. smegmatis ∆perM::perM_mtb_-mCherry::gfp* served as a control. GFP-containing proteins were immunoprecipitated with anti-GFP mAB-agarose beads. Whole-cell lysates (input) and eluates (pull-down) were analyzed by western blotting with anti-GFP or anti-mCherry antibodies. GFP (cyan arrows), GFP-FtsB_mtb_ (red arrows), PerM_mtb_-mCherry (black arrows). (**B**) Reverse pull-down with PerM_mtb_ as ‘bait’ protein from whole-cell protein lysates of *M. smegmatis* WT::*perM_mtb_-gfp* transformed with a plasmid expressing *snap-ftsB_mtb_*. Lysates from *M. smegmatis* WT::*gfp* transformed with a plasmid expressing *snap-ftsB_mtb_* served as a control. Following immunoprecipitation of GFP-containing proteins with anti-GFP magnetic beads, we analyzed whole-cell lysates and eluates with anti-GFP or anti-SNAP antibodies. GFP (cyan arrows), PerM_mtb_-GFP (black arrows), SNAP-FtsB_mtb_ (red arrows). Data in (**A and B**) are representative of two independent experiments.

**Figure 5—figure supplement 2.**
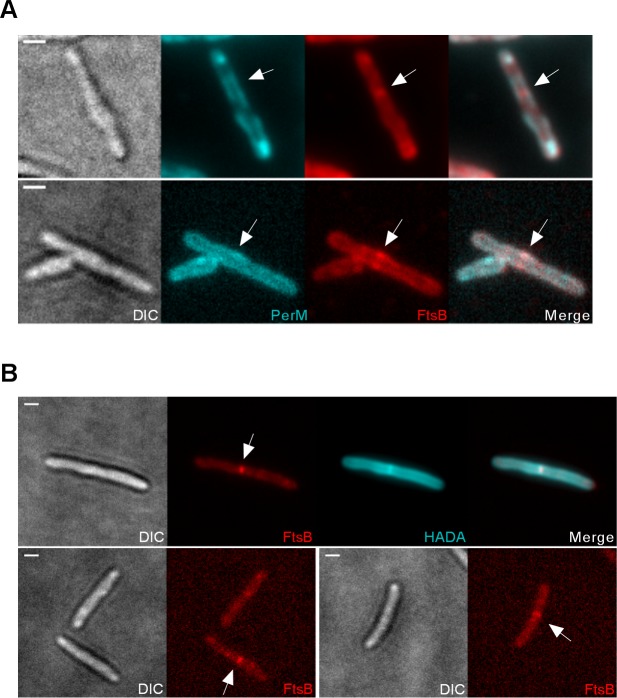
Localization of FtsB to the mid-cell does not depend on the presence of PerM. (**A**) Representative microscopy images of *M. smegmatis ∆perM* expressing PerM_mtb_-GFP and mCherry-FtsB_mtb_ under constitutive promoters. Arrows point to the absence of PerM and presence of FtsB at the mid-cell. Scale bar, 1 µm. (**B**) Representative microscopy images of *M. smegmatis ∆perM* expressing GFP-FtsB_mtb_ under a constitutive promoter. Arrows point to FtsB signals at the mid-cell. Scale bar, 1 µm. Images are representative of two independent experiments. In the top panel, bacteria were incubated in the presence of 1 mM HADA for 6 hr to visualize the septa.

**Figure 5—figure supplement 3.**
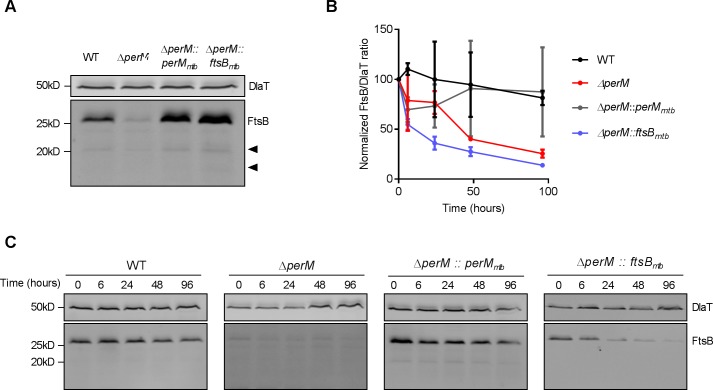
PerM stabilizes FtsB. (**A**) Biological replicates of Figure 5C. (**B**) Quantification of FtsB band intensities from the western blots shown in Figure 5E and Figure 5 - figure supplement 3C. FtsB band intensities were normalized to that of DlaT and the FtsB/DlaT ratios at 0 hour were set to 100% for each strain. Data are means ± SD of biological duplicates. (**C**) Biological replicates of Figure 5E.

### PerM stabilizes FtsB

FtsB protein levels were markedly reduced in Mtb*ΔperM* relative to WT or the complemented mutant, despite similar *ftsB* mRNA levels (Figure 5C,D, Figure 5—figure supplement 3A). We therefore tested whether PerM stabilizes FtsB by monitoring FtsB levels after blocking protein synthesis with chloramphenicol. In both WT Mtb and the complemented mutant, FtsB amounts were stable over 96 hr (Figure 5E, Figure 5—figure supplement 3B,C). In contrast, FtsB abundance decreased over time in *ΔperM*. Because FtsB levels were low in *ΔperM* and thus difficult to assess, we also evaluated FtsB stability in *ΔperM* containing an additional copy of *ftsB* (*ΔperM::ftsB_mtb_*) resulting in increased FtsB levels. In the absence of PerM, the abundance of FtsB still decreased over time. These results demonstrate that PerM stabilizes FtsB.

### PerM is required for normal growth and cell division of Mtb in host-relevant stress conditions

**Figure 6.**
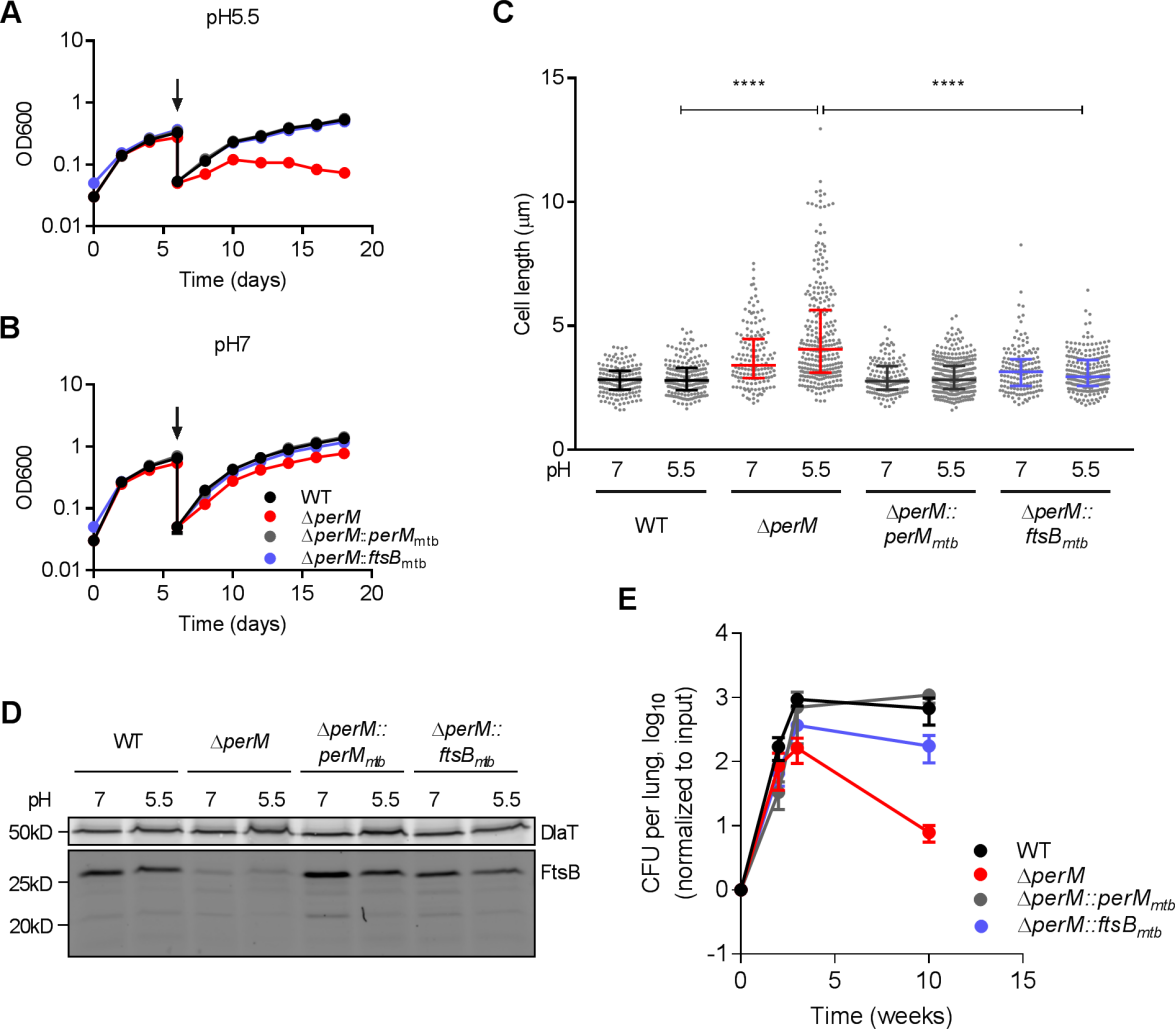
PerM is required for normal growth and cell division of Mtb in host-relevant stress conditions. (**A,B**) Growth curves of Mtb strains sequentially cultured in 7H9 medium adjusted to either pH 5.5 (**A**) or pH 7 (**B**). At day 6 (arrows), the cultures were diluted in the same medium and samples collected for length quantification. Data are means ± SD of three replicates. (**C**) Scatter plots of the cell length of Mtb 6 days post incubation at either pH 5.5 or pH 7. The middle lines represent the medians and the bottom and top lines represent the 25^th^ and 75^th^ percentiles. P-values were calculated using Kruskal-Wallis test and corrected for multiple comparisons. ****, adj-P <0.0001. (**D**) FtsB levels in whole-cell protein lysates assayed by western blotting with anti-FtsB antibody. Whole-cell protein lysates were collected from Mtb cultured in 7H9 medium adjusted to either pH 7 or pH 5.5 for 3 or 6 days. DlaT was used as loading control. (**E**) Bacterial titers in lungs of C57BL/6 mice infected with indicated Mtb strains. Data are means ± SD of four mice and normalized to input. Data in (**A, B and D**) are representative of two independent experiments, and data in (**C and E**) are from one experiment. Figure 6—source data 1.Summary statistics of Figure 6C.

Mtb*ΔperM* was specifically attenuated during the chronic phase of mouse infection following the onset of adaptive immunity when T-cell derived IFN-γ activates macrophages to control Mtb (Flynn et al., 1993). In IFN-γ-activated macrophages, Mtb faces acidic pH, reactive oxygen and nitrogen intermediates and limited amounts of iron and other nutrients (Ehrt and Schnappinger, 2009; Stallings and Glickman, 2010). We reported previously that an Mtb *perM* transposon mutant survived these stresses similarly to WT in vitro (Goodsmith et al., 2015), consistent with our finding that the death rates of *ΔperM* and WT were similar during the chronic phase of mouse infection (Figure 1—figure supplement 2B). Because the growth rate of *ΔperM* was significantly reduced compared to that of WT during chronic infection, we tested whether PerM is required to sustain bacterial replication under host-relevant stress conditions, such as acidic conditions and iron-restricted medium. In contrast to its near normal growth at pH 7, *ΔperM* exhibited a growth defect and elongated cell morphology at pH 5.5 (Figure 6A–C). Ectopically-expressed FtsB reversed the growth defect and cell elongation phenotype, suggesting that both were caused by insufficient amounts of FtsB. Notably, FtsB levels were similar at pH 7 and pH 5.5, suggesting an increased demand for FtsB during growth at low pH (Figure 6D). Growth in iron-depleted medium resulted in phenotypic changes similar to those observed at low pH, and these could also be reversed by ectopic FtsB expression (Figure 6—figure supplement 1). Therefore, in host-relevant stress conditions, PerM is required to maintain FtsB at levels that are sufficient for growth and cell division.

**Figure 6—figure supplement 1.**
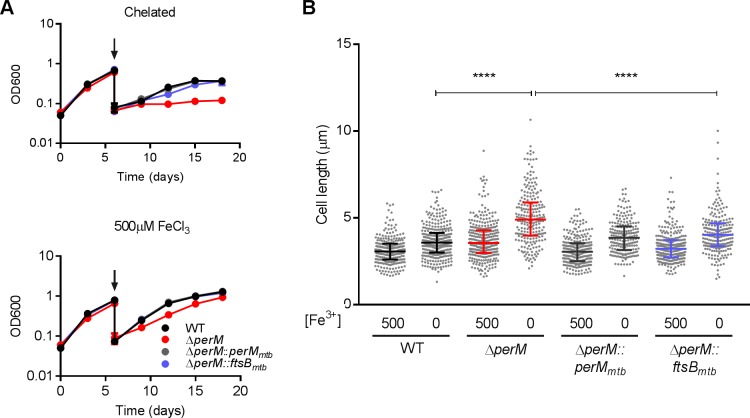
PerM is required for normal growth and cell division of Mtb in low-iron medium. (**A**) Growth curves of Mtb sequentially cultured in chelated Sauton’s medium without adding Fe^3+^ or with addition of 500 µM FeCl_3_. On day 6, we diluted the cultures in the same medium, and collected samples for length quantifications. Data are means ± SD of three replicates. (**B**) Scatter plots of the cell lengths of Mtb after incubation in chelated Sauton’s medium or Sauton’s medium supplemented with 500 µM FeCl_3_ for 6 days. The middle lines represent the medians and the bottom and top lines represent the 25^th^ and 75^th^ percentiles. P-values were calculated using Kruskal-Wallis test and corrected for multiple comparisons. ****, adj-P <0.0001. Data in (**A**) are representative of two independent experiments, and measurements in (**B**) are from one experiment. Figure 6—figure supplement 1—source data 1.Summary statistics of Figure 6—figure supplement 1B.

To extend these findings to the in vivo environment, we infected mice with *ΔperM::ftsB_mtb_* and found that ectopic FtsB expression rescued the persistence defect of *ΔperM* (Figure 6E). Thus, PerM-mediated FtsB stabilization is required for growth and cell division in vivo, specifically in the chronic phase of infection.

Taken together, these results indicate that PerM, a member of the mycobacterial divisome, stabilizes FtsB and maintains it at levels sufficient for cell division in the host-imposed stress conditions that characterize the chronic phase of mouse infection. Our results additionally highlight that Mtb cell division during the chronic phase of mouse infection depends on specific divisome components that differ from those required during the acute phase of infection.

## Discussion

Subclinical persistent infections caused by actively-growing bacteria have been described for multiple bacterial pathogens (Certain et al., 2017; Fisher et al., 2017). In individuals with latent TB infection, bacterial populations are heterogeneous, exhibit differences in metabolic state and susceptibility to antibiotics, and are likely to include actively-replicating subpopulations (Aldridge et al., 2012; Cadena et al., 2017; Esmail et al., 2016; Malherbe et al., 2016). In mice Mtb continues to replicate during chronic infection (Gill et al., 2009), but little is known about the required divisome components. We found that PerM is part of the mycobacterial divisome and facilitates cell division by stabilizing FtsB during chronic mouse infection. FtsB is a member of the divisome in many bacterial species. In *E. coli*, FtsB is associated with two other bitopic membrane proteins, FtsQ and FtsL, and promotes septation and cell constriction (Buddelmeijer and Beckwith, 2004; Glas et al., 2015), (Goehring and Beckwith, 2005; Liu et al., 2015; Tsang and Bernhardt, 2015). Mycobacterial FtsQ, FtsL and FtsB are structurally conserved and functionally similar to their *E. coli* homologs (Jain et al., 2018; Wu et al., 2018); however, regulation of FtsB differs between the two species. In *E. coli*, the stability of FtsB depends on FtsL and vice versa (Gonzalez and Beckwith, 2009), while we found that PerM is required to stabilize FtsB in Mtb; FtsL alone does not protect FtsB from degradation in the absence of PerM. PerM is highly conserved among mycobacteria and other actinobacteria but has no homologs outside of this group, implying its function may be specific to actinobacteria. Within mycobacteria, we have shown that PerM encoded by Mtb and *M. smegmatis* are conserved in function (Figure 4B), but the two proteins differ in essentiality for growth under standard experimental conditions. This difference may be due to a varied demand for FtsB during cell division in the sister species. Immunoprecipitation experiments revealed that PerM physically interacts with FtsB (Table 1, Figure 5—figure supplement 1), although it is not clear whether this represents direct binding, or an interaction mediated by other proteins. Nonetheless, PerM may protect FtsB from recognition or access by an unidentified protease. A similar scenario has been observed in *B. subtilis*, the FtsB homolog DivIC interacts with FtsL, preventing proteolysis of FtsL by the intramembrane protease RasP (Wadenpohl and Bramkamp, 2010). Alternatively, PerM may stabilize FtsB and possibly the FtsQLB complex (Wu et al., 2018) by facilitating protein-protein interactions within the divisome.

What could be the benefit of expressing an inherently unstable protein such as FtsB along with a stabilizing factor such as PerM, as opposed to simply expressing a more stable form of the former? Examples of other cell division proteins suggest that their inherent instability is important for temporal control of cell division. In *B. subtilis*, FtsL degradation by RasP has been proposed to occur after the cell cycle has been completed and the divisome disassembled, thus preventing reassembly of the divisome and subsequent cell division at the wrong time (Wadenpohl and Bramkamp, 2010). In *E. coli*, the concentration of FtsZ has been shown to oscillate during the cell cycle in slow growth conditions and FtsZ is degraded by the ClpXP protease at the end of the cell cycle (Männik et al., 2018). Further, the amplitude of FtsZ oscillation increased in conditions where growth is slowed, indicating that growth conditions may impact the regulation of cell division. In Mtb, the expression of an unstable FtsB along with its stabilizer PerM may allow for fine-tuning of FtsB levels during the cell cycle, which could be important in adaptation to the host environment. Consistent with this hypothesis, *ΔperM* showed growth and cell division defects in stress conditions mimicking the intracellular environment.

The abundance of FtsB was not significantly changed upon exposure of the bacteria to stress despite a marked reduction in cell replication rate. This observation indicates that either FtsB activity is altered under stress conditions or that the divisome formed in Mtb under stress conditions differs from the standard log-phase divisome, displaying an increased demand for FtsB. These hypotheses are supported by studies in mycobacteria that showed alterations in activities or protein-protein interactions of divisome components in response to stress. For example, MarP, a periplasmic protease, interacts more strongly with the peptidoglycan hydrolase RipA in acidic pH, resulting in RipA activation (Botella et al., 2017a). In addition, FtsZ requires interaction with FipA for mid-cell localization and Z-ring formation in *M. smegmatis* during oxidative stress, but not in normal growth conditions (Sureka et al., 2010). Z-ring formation has also been shown to be compromised in intracellular Mtb, likely due to altered FtsZ stability or activity in stresses imposed by the intracellular environment (Chauhan et al., 2006).

The environmental stimuli regulating cell division proteins are often transduced by protein kinases (Molle and Kremer, 2010). In addition to PerM and FtsQ, our co-immunoprecipitation experiment revealed interaction of FtsB with PknH, a Ser/Thr protein kinase involved in regulation of cell division (Sharma et al., 2006; Zheng et al., 2007) (Table 1). Other FtsB interactors include cell division-related proteins such as DacB1, FtsK and PonA1; an ATP-binding protein; membrane proteins; an inositol 1-phosphate synthase; transporters; and proteins of unknown function. Future research will aim to delineate the pathways connecting environmental changes to FtsB regulation in Mtb.

In summary, this work provides new insights into the regulation of cell division in Mtb and its importance for persistence in the host. The cell division and in vivo persistence defects caused by inactivating PerM, a protein dispensable for bacterial replication in standard in vitro growth conditions, reveal that the requirements for cell division differ in vitro between standard growth conditions and host-relevant stress conditions, as well as in vivo between acute and chronic phases of mouse infection. Whether sustained cell replication also facilitates persistence of Mtb in humans is as yet unknown, but evidence for ongoing bacterial transcription and bacterial heterogeneity during latent TB infection support this hypothesis (Esmail et al., 2016; Malherbe et al., 2016). Given the importance of cell division for persistence of Mtb in chronically-infected mice and its potential importance for persistence in latently-infected humans, future work will identify and characterize cell division factors that are specifically required for persistence in vivo. This will not only provide insights into host-pathogen interactions, but also lay the groundwork for potential strategies to combat persistent Mtb infections.

## Materials and methods

**Key resources table:** 

Reagent type (species) or resource	Designation	Source or reference	Identifiers	Additional information
Strain, strain background (*Mycobacterium tuberculosis*)	WT	This work		H37Rv
Strain, strain background (*Mycobacterium tuberculosis*)	*∆perM*	doi: 10.1128/AAC.01334-17		H37Rv
Strain, strain background (*Mycobacterium tuberculosis*)	*∆perM*::*perM_mtb_*	This work		H37Rv *∆perM*::hsp60-*perM_mtb_*
Strain, strain background (*Mycobacterium tuberculosis*)	*∆perM*::*ftsB_mtb_*	This work		H37Rv *∆perM*::hsp60-*ftsB_mtb_*
Strain, strain background (*Mycobacterium tuberculosis*)	WT-pBP10	This work Plasmid information in doi: 10.1038/nm.1915		H37Rv pBP10 plasmid obtained from Dr. David Sherman (University of Washington)
Strain, strain background (*Mycobacterium tuberculosis*)	*∆perM*-pBP10	This work Plasmid information in doi: 10.1038/nm.1915		H37Rv pBP10 plasmid obtained from Dr. David Sherman (University of Washington)
Strain, strain background (*Mycobacterium tuberculosis*)	WT::hsp60-*flag*	This work		H37Rv
Strain, strain background (*Mycobacterium tuberculosis*)	WT::hsp60-*flag-ftsB_mtb_*	This work		H37Rv
Strain, strain background (*Mycobacterium smegmatis*)	WT	This work		mc^2^155
Strain, strain background (*Mycobacterium smegmatis*)	*perM*-DUC	This work		mc^2^155 *ΔperM*::T38S38-P766-9T-*perM_mtb_-gfp*-das+4::TSC10M1-SD2-SSPB
Strain, strain background (*Mycobacterium smegmatis*)	*perM*-DUC-control	This work		mc^2^155 *perM*-DUC + hsp60-empty
Strain, strain background (*Mycobacterium smegmatis*)	*perM*-DUC- *perM_mtb_*	This work		mc^2^155 *perM*-DUC + hsp60-*perM_mtb_-gfp*
Strain, strain background (*Mycobacterium smegmatis*)	*perM*-DUC- *perM_msm_*	This work		mc^2^155 *perM*-DUC + hsp60-*perM_msm_-gfp*
Strain, strain background (*Mycobacterium smegmatis*)	*perM*-DUC-*ftsB_mtb_*	This work		mc^2^155 *perM*-DUC + hsp60-*ftsB_mtb_*
Strain, strain background (*Mycobacterium smegmatis*)	*perM*-DUC-*ftsB_msm_*	This work		mc^2^155 *perM*-DUC + hsp60-*ftsB_msm_*
Strain, strain background (*Mycobacterium smegmatis*)	*ΔperM*::P38-*perM_mtb_-mcherry*:: hsp60-*gfp-ftsB_mtb_*	This work		mc^2^155
Strain, strain background (*Mycobacterium smegmatis*)	*ΔperM*::P38-*perM_mtb_-mcherry*:: hsp60-*gfp*	This work		mc^2^155
Strain, strain background (*Mycobacterium smegmatis*)	WT::P38-*gfp +* hsp60-*snap-ftsB_mtb_*	This work		mc^2^155
Strain, strain background (*Mycobacterium smegmatis*)	WT::P38-*perM_mtb_-gfp +* hsp60-*snap-ftsB_mtb_*	This work		mc^2^155
Strain, strain background (*Mycobacterium smegmatis*)	*ΔperM*::P38-*perM_mtb_-gfp*::hsp60-*mcherry-ftsB_mtb_*	This work		mc^2^155
Strain, strain background (*Mycobacterium smegmatis*)	*ΔperM*::P38-*gfp-ftsB_mtb_*	This work		mc^2^155
Strain, strain background (*Mycobacterium smegmatis*)	*ΔperM*::hsp60-*gfp-ftsB_mtb_*	This work		mc^2^155
Antibody	Anti-Flag M2 affinity gel	Sigma	A2220	
Antibody	Anti-GFP mAb-agarose beads	MBL	D153-8	
Antibody	Anti-GFP mAb-magnetic beads	MBL	D153-11	
Antibody	Anti-FtsB (Rabbit polyclonal)	GenScript	U1550BG270	(1:1000)
Antibody	Anti-DlaT (Rabbit polyclonal)	doi: 10.1126/science.1067798		Antibody from Drs. Carl Nathan and Ruslana Bryk (Weill Cornell Medicine) (1:10000)
Antibody	Anti-GFP (Mouse monoclonal)	Clontech	632380	(1:8000)
Antibody	Anti-mCherry (Mouse monoclonal)	Clontech	632543	(1:1000)
Antibody	Anti-SNAP (Rabbit polyclonal)	New England BioLabs	P9310S	(1:1000)
Antibody	donkey anti-rabbit 680	LI-COR Biosciences	926–68023	(1:10000)
Antibody	donkey anti-rabbit 800	LI-COR Biosciences	926–32213	(1:10000)
Antibody	goat anti-mouse 800	LI-COR Biosciences	926–32210	(1:5000)
Peptide, recombinant protein	FLAG peptide	Sigma	F3290	(100 ng/µl)
Software, algorithm	Prism 7	Graphpad		
Software, algorithm	ImageJ	doi: 10.1038/nmeth.2089		
Software, algorithm	Icy	doi:10.1038/nmeth.2075		
Software, algorithm	Scaffold 4			
Other	SYTO 13 Green Fluorescent Nucleic Acid Stain	Thermo Fisher Scientific	S7575	(5 μM)
Other	HADA	doi: 10.1002/anie.201206749;doi: 10.1038/nprot.2014.197		(1 mM) Synthesized by the Chemical Synthesis Core Facility at MSKCC
Other	NADA	doi: 10.1002/anie.201206749;doi: 10.1038/nprot.2014.197		(1 mM) Synthesized by the Chemical Synthesis Core Facility at MSKCC

### Bacterial culture conditions

*M. smegmatis* mc^2^155 and derivative strains were cultured in Middlebrook 7H9 medium (BD Difco) containing 0.2% glycerol and 0.05% Tween 80 or Middlebrook 7H10 agar (BD Difco) containing 0.5% glycerol. *M. tuberculosis* H37Rv and derivative strains were cultured in Middlebrook 7H9 medium containing 0.2% glycerol, 0.2% dextrose, 0.5% BSA (Roche), 0.05% Tween 80 or tyloxapol, and 0.085% NaCl ﻿or Middlebrook 7H10 agar containing 10% OADC supplement (BD) and 0.5% glycerol. Selection antibiotics were used at the following concentrations: ﻿ hygromycin (50 μg/ml), zeocin (25 μg/ml), kanamycin (25 μg/ml) or streptomycin (20 μg/ml).

### Mutant construction

The Mtb*ΔperM* mutant was constructed by allelic exchange using a recombineering approach as previously described (Gee et al., 2012). The *ΔperM* strain was complemented by introducing a copy of *perM*, expressed under the control of the hsp60 promoter, into the attL5 site of the Mtb genome. The *ΔperM::ftsB* mutant was constructed by integrating a copy of *ftsB*, expressed under the control of the hsp60 promoter, into the attL5 site of the Mtb genome. The *M. smegmatis perM*-DUC mutant was generated as described (Schnappinger et al., 2015). Complementation with *ftsB* was either on a multicopy plasmid or integrated in the chromosomal attL5 site under control of the hsp60 promoter. For FtsB_mtb_ fusion proteins, N-terminal GFP, mCherry, Flag or SNAP tags were introduced to FtsB_mtb_ by PCR. PerM_mtb_ fusion proteins were cloned by fusing mCherry or GFP to the C-terminus of PerM_mtb_. The genotypes of all strains are reported in the key resources table.

### Mouse infections

Female 7–8 week old C57BL/6 mice (Jackson Laboratory) were infected using an inhalation exposure system (Glas-Col) with mid-log phase Mtb culture delivering approximately 10^2^ or 10^3^ bacilli per mouse. At each time point, lungs were homogenized in PBS and bacteria were enumerated by plating serially diluted homogenates on 7H10 agar.

### Replication clock

Experiments were performed as described previously (Gill et al., 2009). Mtb strains were transformed with the ‘replication clock’ plasmid pBP10. For in vitro experiments, cultures were maintained in log phase by sub-culturing every 3 days for ~20 generations in the absence of antibiotics. The plasmid carriage of each Mtb strain was calculated as the number of CFU on 7H10 agar with 30 μg/ml kanamycin divided by the number of CFU on non-selective plates. Segregation rates (s) and errors were calculated using mathematical equations developed by Gill et al. (2009) and the error propagation equation. We determined the plasmid segregation constants for WT (S_WT_ = 0.1904 ± 0.0516) and *ΔperM* (S*_Δ_*=0.1586 ± 0.0349) before measuring cell replication rates in vivo.

For in vivo experiments, cell replication rates (r) were calculated as the slopes of the regression lines fitted through plots of ln [f(t)] versus t and multiplied with (−1/s), where s is the segregation constant determined in vitro. Death rates were calculated by subtracting slope N from r. Doubling time equals ln (2)/r. The errors of both death rate and doubling time were calculated using error propagation equations. The cumulative bacterial burden (CBB) was calculated using mathematical models developed by Gill et al. (2009).

### In situ Mtb cell length quantification

Mice were infected with log-phase Mtb by aerosol to deliver ~10^3^ bacilli per mouse. At each time point, lungs were isolated, and the upper left lung lobes were fixed in 10% buffered formalin for 48 hr prior to removal from BSL-3 containment. The lung lobes were subsequently embedded in paraffin, sectioned and stained with acid-fast staining. Slides were blinded before microscopy and the identity of the samples was revealed after length quantifications were completed. Approximately 40 lung sections were examined for each time point with each strain. Tissue slides were visualized in bright-field using Olympus BX60 microscope with a 100x oil objective and images were captured with Olympus DP71 digital camera. Bacteria in the focal plane of the sections were imaged and their cell lengths were quantified using ImageJ (Schneider et al., 2012). The remaining lung lobes were homogenized in PBS and cultured on 7H10 agar to determine CFU.

### High-resolution microscopy

Microscopy imaging was performed with the same methods and equipment as previously described (Botella et al., 2017b). For length measurements, Mtb or *M. smegmatis* cultures were collected at indicated time points and washed with PBS containing 0.05% Tween 80 (PBST). The Mtb samples were fixed with 4% paraformaldehyde for 4 hr prior to removal from BSL-3 containment. Single cell suspensions were prepared by centrifugation at 800 rpm for 10 min. After spreading on soft agar pads, bacteria were visualized with appropriate filter sets. For peptidoglycan labeling, 1 mM HCC‐amino‐D‐alanine (HADA) or NBD‐amino‐D‐alanine (NADA) was added to *M. smegmatis* cultures as indicated. Aliquots were removed, washed three times with PBST, and then fixed with 4% paraformaldehyde for 30 min before microscopy. The nucleoid staining was performed by incubating *M. smegmatis* cultures with 5 μM SYTO 13 for 5 min. Images were analyzed with ImageJ and Icy (de Chaumont et al., 2012; Schneider et al., 2012).

For time lapse microscopy experiments, a drop of *M. smegmatis* culture (5 ~ 10 μl) was placed on a glass bottom microwell dish (MatTek, 35 mm, 14 mm microwell), and 1% low melting point agarose (UltraPure, Invitrogen) prepared in 7H9 broth was added gently to the dish until it covered the bacteria drop. Agarose was left to solidify prior to microscopy. Cells were visualized by fluorescence microscopy and snapshots were captured every 15 min. We analyzed the time lapse movies with ImageJ (Schneider et al., 2012). First, the fluorescence intensities were measured lengthwise across a single cell at every time point, from which we determined the maximum fluorescence intensities of the middle 1/2 of the cell. Next, each time point was interpolated to 1/10 of the cell cycle of each cell to allow many bacterial cells to be averaged consistently.

### Forward genetic screen

A total of 10^8^ CFU of Msm *perM*-DUC mutant were transformed with an Mtb genomic library generated as described (Niederweis et al., 1999). Bacteria were cultured on 7H10 agar with 400 ng/ml atc and 50 µg/ml hygromycin. Colonies were picked and lysed in 20 µl ice-cold 10% glycerol and transformed into 100 µl *E. coli* top10 competent cells by electroporation. The transformants were selected on LB agar containing 200 µg/ml hygromycin. Plasmids were isolated and sequenced to identify Mtb DNA fragments.

### Immunoprecipitation and mass spectrometry

150 ml of Mtb culture in mid-log phase was pelleted and washed twice with PBST. Cells were resuspended in 600 μl lysis buffer (50 mM Tris-HCl, 50 mM NaCl, pH 7.4) containing protease inhibitor cocktail (Roche) and subjected to mechanical lysis with 0.1 zyrconia beads in a homogenizer. After adding 1% dodecyl maltoside, we incubated the protein lysates on ice for 2 hr. Lysates were clarified by centrifugation for 2 min at 7,500 g. 50 μl anti-Flag M2 affinity beads (Sigma, A2220) were added to supernatants and incubated overnight shaking at 4°C. Beads were collected by centrifugation and washed 5 times with PBS. Proteins bound to beads were eluted by incubation with 100 ng/μl flag peptide (Sigma) 1 hr shaking at 4°C. Samples were boiled in Laemmli sample buffer (Bio-Rad) for 10 min prior to removal from BSL-3 containment. Peptides in the eluates were identified by mass spectrometry. Results were analyzed using Scaffold 4 software. Non-specific binding peptides were removed from the results by setting the filter of ‘Total Spectrum Count’ of each replicate to ‘<2’ in the control samples and ‘>3’ in Flag-FtsB samples. Hits were ranked based on ‘Sum Total Spectrum Count’ of the Flag-FtsB samples. The ‘Sum Total Spectrum Count’ is the sum of two independent biological replicates. Hits were assigned utilizing publicly available databases PATRIC (https://www.patricbrc.org/) and FLUTE (http://orca2.tamu.edu/U19/). For pulldown validations, 150 ml M. *smegmatis* was grown to mid-log phase and protein lysates were collected as described above. Protein lysate (10–30 mg) was incubated with 25 μl anti-GFP mAb-agarose beads or 50 μl anti-GFP mAb-magnetic beads (MBL) overnight shaking at 4°C. After incubation, beads were collected by centrifugation or using a DynaMag-Magnet 2 rack (Thermo Fisher scientific) and washed three times with PBS. Proteins bound to beads were eluted by boiling in Laemmli sample buffer for 10 min and analyzed by western blotting.

### Antibodies and western blots

Rabbit polyclonal antibody for FtsB was generated by GenScript. DlaT antibody (Bryk et al., 2002) was a gift from R. Bryk and C. Nathan at Weill Cornell Medicine. GFP (Clontech #632380), mCherry (Clontech #632543) and SNAP (NEB #P9310S) antibodies are commercially available. All secondary antibodies were purchased from LI-COR biosciences.

For western blots, protein lysates were prepared by mechanical lysis with 0.1 mm zirconia beads, followed by 2 hr incubation on ice with 1% dodecyl maltoside. Unbroken bacterial cells and beads were removed by centrifugation. Mtb protein lysates were filtered using 0.22 µm spin-X columns (Corning) prior to removal from BSL3 containment. Protein lysates were separated using SDS-PAGE and transferred to a nitrocellulose membrane. For FtsB analysis, membrane was cut at 37kD and incubated with either FtsB antibody or DlaT antibody. After washing and incubation with secondary antibodies, proteins were visualized using Odyssey Infrared Imaging System (LI-COR Biosciences).

### Analysis of protein stability

Chloramphenicol (Sigma) stock was prepared at 100 mg/ml in ethanol and added to mid-log phase Mtb cultures at a final concentration of 20 μg/ml. At each time point, 30 ml Mtb cultures were removed and protein lysates collected as described above. Stability of FtsB was analyzed by western blotting.

### Mtb growth assays

Mtb was grown to mid-log phase in Middlebrook 7H9 medium prior to each experiment. The precultures were pelleted at 4000 rpm for 10 min and washed once in PBS, then diluted to OD 0.05 in assay media. For growth curves in acidic or low iron conditions, Mtb cultures were diluted to OD 0.05 in assay media at day 6. Conditions for the growth curves were as follows: Magnesium-free Sauton’s media was prepared as described (Goodsmith et al., 2015). Magnesium was supplemented in the form of MgCl_2_ at the indicated concentrations. 7H9 medium at pH 5.5 or pH 7 were prepared by adjusting 7H9 broth containing 0.2% glycerol, 0.2% dextrose, 0.5% fatty-acid-free BSA (Roche), 0.085% NaCl, 1.95% MES, and 0.05% tyloxapol to pH as indicated. 0.005% sodium oleate was supplemented every other day. Medium with defined iron concentrations was prepared by chelating Sauton’s medium (0.4% L-asparagine, 0.27% sodium citrate tribasic dehydrate, 0.015% citric acid, 0.05% potassium phosphate dibasic, and 6% glycerol) with 1% Chelex-100 (sodium form, Bio-Rad) twice for 24 hr. Subsequently Chelex-100 was removed by filtration and 0.05% magnesium sulfate heptahydrate, 5 μM zinc sulfate, 0.1% ethanol, and 0.05% tyloxapol was added to the medium and the medium was adjusted to pH 7.4. Iron was supplemented in the form of FeCl_3_ at the indicated concentrations.

### Statistical analysis

All statistical analyses were performed using Prism 7 (GraphPad Software) with the statistical tests indicated in figure legends and the corresponding two-tailed P-values reported. *, p<0.05, **, p<0.01, ***, p<0.001 and ****, p<0.0001 were considered significant results.

### Materials and correspondence

Further information and requests for resources and reagents should be directed to the corresponding author Sabine Ehrt (sae2004@med.cornell.edu).

## Data Availability

All data generated or analyzed during this study are included in the manuscript and supporting files. Source data files have been provided for Figures 1, 2, 3, 4 and 6 and Table 1.

## Additional files

Transparent reporting form

## References

[bib1] Aldridge BB, Fernandez-Suarez M, Heller D, Ambravaneswaran V, Irimia D, Toner M, Fortune SM (2012). Asymmetry and aging of mycobacterial cells lead to variable growth and antibiotic susceptibility. Science.

[bib2] Behr MA, Edelstein PH, Ramakrishnan L (2018). Revisiting the timetable of tuberculosis. BMJ.

[bib3] Botella H, Vaubourgeix J, Lee MH, Song N, Xu W, Makinoshima H, Glickman MS, Ehrt S (2017a). *Mycobacterium tuberculosis* protease MarP activates a peptidoglycan hydrolase during acid stress. The EMBO Journal.

[bib4] Botella H, Yang G, Ouerfelli O, Ehrt S, Nathan CF, Vaubourgeix J (2017b). Distinct Spatiotemporal Dynamics of Peptidoglycan Synthesis between *Mycobacterium smegmatis* and *Mycobacterium tuberculosis*. mBio.

[bib5] Bryk R, Lima CD, Erdjument-Bromage H, Tempst P, Nathan C (2002). Metabolic enzymes of mycobacteria linked to antioxidant defense by a thioredoxin-like protein. Science.

[bib6] Buddelmeijer N, Judson N, Boyd D, Mekalanos JJ, Beckwith J (2002). YgbQ, a cell division protein in Escherichia coli and Vibrio cholerae, localizes in codependent fashion with FtsL to the division site. PNAS.

[bib7] Buddelmeijer N, Beckwith J (2004). A complex of the Escherichia coli cell division proteins FtsL, FtsB and FtsQ forms independently of its localization to the septal region. Molecular Microbiology.

[bib8] Cadena AM, Fortune SM, Flynn JL (2017). Heterogeneity in tuberculosis. Nature Reviews Immunology.

[bib9] Carson MJ, Barondess J, Beckwith J (1991). The FtsQ protein of Escherichia coli: membrane topology, abundance, and cell division phenotypes due to overproduction and insertion mutations. Journal of Bacteriology.

[bib10] Certain LK, Way JC, Pezone MJ, Collins JJ (2017). Using engineered Bacteria to characterize infection dynamics and antibiotic effects in Vivo. Cell Host & Microbe.

[bib11] Chauhan A, Madiraju MV, Fol M, Lofton H, Maloney E, Reynolds R, Rajagopalan M (2006). Mycobacterium tuberculosis cells growing in macrophages are filamentous and deficient in FtsZ rings. Journal of Bacteriology.

[bib12] de Chaumont F, Dallongeville S, Chenouard N, Hervé N, Pop S, Provoost T, Meas-Yedid V, Pankajakshan P, Lecomte T, Le Montagner Y, Lagache T, Dufour A, Olivo-Marin JC (2012). Icy: an open bioimage informatics platform for extended reproducible research. Nature Methods.

[bib13] Ehrt S, Schnappinger D, Rhee KY (2018). Metabolic principles of persistence and pathogenicity in Mycobacterium tuberculosis. Nature Reviews Microbiology.

[bib14] Ehrt S, Schnappinger D (2009). Mycobacterial survival strategies in the phagosome: defence against host stresses. Cellular Microbiology.

[bib15] Esmail H, Lai RP, Lesosky M, Wilkinson KA, Graham CM, Coussens AK, Oni T, Warwick JM, Said-Hartley Q, Koegelenberg CF, Walzl G, Flynn JL, Young DB, Barry Iii CE, O'Garra A, Wilkinson RJ (2016). Characterization of progressive HIV-associated tuberculosis using 2-deoxy-2-[18F]fluoro-D-glucose positron emission and computed tomography. Nature Medicine.

[bib16] Fisher RA, Gollan B, Helaine S (2017). Persistent bacterial infections and persister cells. Nature Reviews Microbiology.

[bib17] Flynn JL, Chan J, Triebold KJ, Dalton DK, Stewart TA, Bloom BR (1993). An essential role for interferon gamma in resistance to Mycobacterium tuberculosis infection. Journal of Experimental Medicine.

[bib18] Gee CL, Papavinasasundaram KG, Blair SR, Baer CE, Falick AM, King DS, Griffin JE, Venghatakrishnan H, Zukauskas A, Wei JR, Dhiman RK, Crick DC, Rubin EJ, Sassetti CM, Alber T (2012). A phosphorylated pseudokinase complex controls cell wall synthesis in mycobacteria. Science Signaling.

[bib19] Gill WP, Harik NS, Whiddon MR, Liao RP, Mittler JE, Sherman DR (2009). A replication clock for Mycobacterium tuberculosis. Nature Medicine.

[bib20] Glas M, van den Berg van Saparoea HB, McLaughlin SH, Roseboom W, Liu F, Koningstein GM, Fish A, den Blaauwen T, Heck AJ, de Jong L, Bitter W, de Esch IJ, Luirink J (2015). The soluble periplasmic domains of *Escherichia coli* Cell Division Proteins FtsQ/FtsB/FtsL Form a Trimeric Complex with Submicromolar Affinity. Journal of Biological Chemistry.

[bib21] Glickman MS, Jacobs WR (2001). Microbial pathogenesis of Mycobacterium tuberculosis: dawn of a discipline. Cell.

[bib22] Goehring NW, Beckwith J (2005). Diverse paths to midcell: assembly of the bacterial cell division machinery. Current Biology.

[bib23] Gonzalez MD, Beckwith J (2009). Divisome under construction: distinct domains of the small membrane protein FtsB are necessary for interaction with multiple cell division proteins. Journal of Bacteriology.

[bib24] Goodsmith N, Guo XV, Vandal OH, Vaubourgeix J, Wang R, Botella H, Song S, Bhatt K, Liba A, Salgame P, Schnappinger D, Ehrt S (2015). Disruption of an M. tuberculosis membrane protein causes a magnesium-dependent cell division defect and failure to persist in mice. PLOS Pathogens.

[bib25] Guzman L-M, Barondess JJ, Beckwith J (1992). FtsL, an Essential Cytoplasmic Membrane Protein Involved in Cell Division in *Escherichia coli*. Journal of Bacteriology.

[bib26] Jain P, Malakar B, Khan MZ, Lochab S, Singh A, Nandicoori VK (2018). Delineating FtsQ-mediated regulation of cell division in *Mycobacterium tuberculosis*. Journal of Biological Chemistry.

[bib27] Katis VL, Wake RG, Harry EJ (2000). Septal localization of the membrane-bound division proteins of Bacillus subtilis DivIB and DivIC is codependent only at high temperatures and requires FtsZ. Journal of Bacteriology.

[bib28] Kieser KJ, Rubin EJ (2014). How sisters grow apart: mycobacterial growth and division. Nature Reviews Microbiology.

[bib29] Kim JH, O'Brien KM, Sharma R, Boshoff HI, Rehren G, Chakraborty S, Wallach JB, Monteleone M, Wilson DJ, Aldrich CC, Barry CE, Rhee KY, Ehrt S, Schnappinger D (2013). A genetic strategy to identify targets for the development of drugs that prevent bacterial persistence. PNAS.

[bib30] Kureisaite-Ciziene D, Varadajan A, McLaughlin SH, Glas M, Montón Silva A, Luirink R, Mueller C, den Blaauwen T, Grossmann TN, Luirink J, Löwe J (2018). Structural analysis of the interaction between the bacterial cell division proteins FtsQ and FtsB. mBio.

[bib31] Kuru E, Hughes HV, Brown PJ, Hall E, Tekkam S, Cava F, de Pedro MA, Brun YV, VanNieuwenhze MS (2012). In Situ probing of newly synthesized peptidoglycan in live Bacteria with fluorescent D-amino acids. Angewandte Chemie International Edition.

[bib32] Kuru E, Tekkam S, Hall E, Brun YV, Van Nieuwenhze MS (2015). Synthesis of fluorescent D-amino acids and their use for probing peptidoglycan synthesis and bacterial growth in situ. Nature Protocols.

[bib33] Levin PA, Losick R (1994). Characterization of a cell division gene from Bacillus subtilis that is required for vegetative and sporulation septum formation. Journal of Bacteriology.

[bib34] Liu B, Persons L, Lee L, de Boer PA (2015). Roles for both FtsA and the FtsBLQ subcomplex in FtsN-stimulated cell constriction in *Escherichia coli*. Molecular Microbiology.

[bib35] Logsdon MM, Aldridge BB (2018). Stable regulation of cell cycle events in mycobacteria: insights from inherently heterogeneous bacterial populations. Frontiers in Microbiology.

[bib36] Malherbe ST, Shenai S, Ronacher K, Loxton AG, Dolganov G, Kriel M, Van T, Chen RY, Warwick J, Via LE, Song T, Lee M, Schoolnik G, Tromp G, Alland D, Barry CE, Winter J, Walzl G, Lucas L, Spuy GV, Stanley K, Thiart L, Smith B, Du Plessis N, Beltran CG, Maasdorp E, Ellmann A, Choi H, Joh J, Dodd LE, Allwood B, Koegelenberg C, Vorster M, Griffith-Richards S, Catalysis TB–Biomarker Consortium (2016). Persisting positron emission tomography lesion activity and Mycobacterium tuberculosis mRNA after tuberculosis cure. Nature Medicine.

[bib37] Männik J, Walker BE, Männik J (2018). Cell cycle-dependent regulation of FtsZ in *Escherichia coli* in slow growth conditions. Molecular Microbiology.

[bib38] Molle V, Kremer L (2010). Division and cell envelope regulation by ser/Thr phosphorylation: *Mycobacterium* shows the way. Molecular Microbiology.

[bib39] Niederweis M, Ehrt S, Heinz C, Klöcker U, Karosi S, Swiderek KM, Riley LW, Benz R (1999). Cloning of the mspA gene encoding a porin from Mycobacterium smegmatis. Molecular Microbiology.

[bib40] Pai M, Behr MA, Dowdy D, Dheda K, Divangahi M, Boehme CC, Ginsberg A, Swaminathan S, Spigelman M, Getahun H, Menzies D, Raviglione M (2016). Tuberculosis. Nature Reviews Disease Primers.

[bib41] Rego EH, Audette RE, Rubin EJ (2017). Deletion of a mycobacterial divisome factor collapses single-cell phenotypic heterogeneity. Nature.

[bib42] Schnappinger D, O'Brien KM, Ehrt S, Gillespie S. H (2015). Construction of Conditional Knockdown Mutants in Mycobacteria. Antibiotic Resistance Protocols.

[bib43] Schneider CA, Rasband WS, Eliceiri KW (2012). NIH image to ImageJ: 25 years of image analysis. Nature Methods.

[bib44] Sharma K, Gupta M, Pathak M, Gupta N, Koul A, Sarangi S, Baweja R, Singh Y (2006). Transcriptional control of the mycobacterial embCAB operon by PknH through a regulatory protein, EmbR, in vivo. Journal of Bacteriology.

[bib45] Stallings CL, Glickman MS (2010). Is Mycobacterium tuberculosis stressed out? A critical assessment of the genetic evidence. Microbes and Infection.

[bib46] Sureka K, Hossain T, Mukherjee P, Chatterjee P, Datta P, Kundu M, Basu J (2010). Novel role of phosphorylation-dependent interaction between FtsZ and FipA in mycobacterial cell division. PLOS ONE.

[bib47] Taguchi A, Welsh MA, Marmont LS, Lee W, Sjodt M, Kruse AC, Kahne D, Bernhardt TG, Walker S (2019). FtsW is a peptidoglycan polymerase that is functional only in complex with its cognate penicillin-binding protein. Nature Microbiology.

[bib48] Tsang MJ, Bernhardt TG (2015). A role for the FtsQLB complex in Cytokinetic ring activation revealed by an ftsL allele that accelerates division. Molecular Microbiology.

[bib49] Wadenpohl I, Bramkamp M (2010). DivIC Stabilizes FtsL against RasP Cleavage. Journal of Bacteriology.

[bib50] Wu KJ, Zhang J, Baranowski C, Leung V, Rego EH, Morita YS, Rubin EJ, Boutte CC (2018). Characterization of conserved and novel septal factors in *Mycobacterium smegmatis*. Journal of Bacteriology.

[bib51] Xu W, DeJesus MA, Rücker N, Engelhart CA, Wright MG, Healy C, Lin K, Wang R, Park SW, Ioerger TR, Schnappinger D, Ehrt S (2017). Chemical genetic interaction profiling reveals determinants of intrinsic antibiotic resistance in Mycobacterium tuberculosis. Antimicrobial Agents and Chemotherapy.

[bib52] Zheng X, Papavinasasundaram KG, Av-Gay Y (2007). Novel substrates of Mycobacterium tuberculosis PknH ser/Thr kinase. Biochemical and Biophysical Research Communications.

