# Supplementary material for: Persistent Mycobacterium tuberculosis infection in mice requires PerM for successful cell division
Source: eLife. 2019 Nov 21;8:e49570. doi: 10.7554/eLife.49570 (PMC6872210; doi:10.7554/eLife.49570)
Supplement: Figure 1—source data 1. [file elife-49570-fig1-data1.pdf]

Figure 1 – Source data 1. Summary statistics of Figure 1D

	**Week 2**		**Week 3**		**Week 4**		**Week 5**	
**(μm)**	**WT**	***ΔperM***	**WT**	***ΔperM***	**WT**	***ΔperM***	**WT**	***ΔperM***
Sample size	176	165	184	199	197	185	176	137
Minimum	1.519	1.181	1.281	1.696	1.740	1.584	1.762	2.396
25th Percentile	2.230	2.318	2.203	2.905	2.689	3.182	2.908	3.469
Median	2.687	2.758	2.654	3.605	3.337	3.961	3.510	4.030
75th percentile	3.255	3.228	3.416	4.460	3.943	4.838	4.295	5.111
Maximum	5.423	5.334	6.188	6.506	6.243	7.663	6.538	8.515
95% confidence interval	2.663-2.874	2.735-2.982	2.766-3.036	3.579-3.864	3.271-3.517	3.861-4.181	3.541-3.840	4.108-4.491
