# Supplementary material for: Persistent Mycobacterium tuberculosis infection in mice requires PerM for successful cell division
Source: eLife. 2019 Nov 21;8:e49570. doi: 10.7554/eLife.49570 (PMC6872210; doi:10.7554/eLife.49570)
Supplement: Figure 2—source data 1. [file elife-49570-fig2-data1.pdf]

Figure 2 – Source data 1. Summary statistics of Figure 2B

	**WT**		***perM*-DUC**	
**(μm)**	**No atc**	**+ atc**	**No atc**	**+ atc**
Sample size	284	282	284	265
Minimum	1.636	2.404	1.916	4.520
25th Percentile	4.147	3.920	3.611	8.865
Median	5.105	4.992	4.460	11.10
75th percentile	6.030	5.966	5.557	14.82
Maximum	12.26	11.01	11.53	30.20
95% confidence interval	4.991-5.315	4.882-5.223	4.463-4.805	11.64-12.75
