# Supplementary material for: Persistent Mycobacterium tuberculosis infection in mice requires PerM for successful cell division
Source: eLife. 2019 Nov 21;8:e49570. doi: 10.7554/eLife.49570 (PMC6872210; doi:10.7554/eLife.49570)
Supplement: Figure 3—source data 1. [file elife-49570-fig3-data1.pdf]

Figure 3 – Source data 1. Summary statistics of Figure 3C

	perM-DUC			
Time (hours)	1.5	1.5	4	10
**(µm)**	**No atc**	**+ atc**	**+ atc**	**+ atc**
Sample size	291	341	307	319
Minimum	1.897	1.991	2.412	2.311
25th Percentile	3.144	3.629	4.166	4.748
Median	3.79	4.598	5.191	6.948
75th percentile	4.657	5.63	6.198	10.25
Maximum	9.274	11.27	14.53	23.29
95% confidence interval	3.871 - 4.12	4.619 - 4.938	5.159 - 5.504	7.585 - 8.497
