# Supplementary material for: Persistent Mycobacterium tuberculosis infection in mice requires PerM for successful cell division
Source: eLife. 2019 Nov 21;8:e49570. doi: 10.7554/eLife.49570 (PMC6872210; doi:10.7554/eLife.49570)
Supplement: Figure 3—source data 2. [file elife-49570-fig3-data2.pdf]

Figure 3 – Source data 2. Summary of Figure 3D and 3E

***perM*-DUC 1.5 hours (No atc)**	% of bacteria with septa labeled with indicated D-alanine			Sample size
	only NADA	only HADA	HADA + NADA	
Exp 1	20.54	0.00	0.71	1553
Exp 2	23.10	0.22	0.45	896
Exp 3	27.66	0.00	0.45	441

***perM*-DUC 1.5 hours (+ atc)**	% of bacteria with septa labeled with indicated D-alanine			Sample size
	only NADA	only HADA	HADA + NADA	
Exp 1	13.82	0.16	1.79	1845
Exp 2	12.43	0.00	0.90	1327
Exp 3	17.60	0.00	0.56	892

***perM*-DUC 4 hours (+ atc)**	% of bacteria with septa labeled with indicated D-alanine			Sample size
	only NADA	only HADA	HADA + NADA	
Exp 1	10.53	0.23	3.51	1766
Exp 2	13.85	0.38	4.27	1054
Exp 3	13.31	0.00	7.62	879

***perM*-DUC 10 hours (+ atc)**	% of bacteria with septa labeled with indicated D-alanine			
	only NADA	only HADA	HADA + NADA	Sample size
Exp 1	14.25	0.48	9.34	835
Exp 2	14.18	0.08	6.88	1178
Exp 3	19.34	0.17	8.48	1179
