# Supplementary material for: Persistent Mycobacterium tuberculosis infection in mice requires PerM for successful cell division
Source: eLife. 2019 Nov 21;8:e49570. doi: 10.7554/eLife.49570 (PMC6872210; doi:10.7554/eLife.49570)
Supplement: Figure 4—source data 1. [file elife-49570-fig4-data1.pdf]

Figure 4 – Source data 1. Summary statistics of Figure 4B

	**empty**		***perM**$_{mtb}$*		***perM**$_{msm}$*		***ftsB**$_{mtb}$*		***ftsB**$_{msm}$*	
**(μm)**	**No atc**	**+ atc**	**No atc**	**+ atc**	**No atc**	**+ atc**	**No atc**	**+ atc**	**No atc**	**+ atc**
Sample size	100	100	100	100	100	100	100	100	100	100
Minimum	3.066	4.206	3.064	2.946	3.067	2.935	4.028	2.797	2.726	2.788
25th Percentile	4.468	8.229	4.518	4.381	4.595	4.602	4.938	4.686	4.866	4.719
Median	5.422	10.65	5.230	5.272	5.291	5.156	5.466	5.508	5.632	5.412
75th percentile	6.114	13.51	5.964	5.839	6.385	5.935	6.105	6.414	6.622	6.422
Maximum	9.263	28.73	11.31	7.320	9.329	8.092	8.543	8.599	10.61	9.128
95% confidence interval	5.201-5.666	10.65-12.53	5.078-5.558	4.922-5.340	5.280-5.808	5.053-5.427	5.390-5.733	5.313-5.787	5.573-6.132	5.339-5.875
