# Supplementary material for: Persistent Mycobacterium tuberculosis infection in mice requires PerM for successful cell division
Source: eLife. 2019 Nov 21;8:e49570. doi: 10.7554/eLife.49570 (PMC6872210; doi:10.7554/eLife.49570)
Supplement: Figure 4—source data 2. [file elife-49570-fig4-data2.pdf]

Figure 4 – Source data 2. Summary statistics of Figure 4D

(μm)	WT				*ΔperM*			
**[Mg^{2+}] μM**	**25**	**100**	**250**	**2000**	**25**	**100**	**250**	**2000**
Sample size	222	163	206	234	162	204	162	206
Minimum	1.619	1.694	1.571	1.451	2.245	2.354	1.954	1.950
25th Percentile	2.781	2.803	2.830	2.750	6.482	4.347	3.827	2.876
Median	3.293	3.198	3.430	3.214	8.128	5.771	4.898	3.588
75th percentile	3.943	3.920	4.138	3.722	10.15	7.185	6.014	4.184
Maximum	6.418	5.430	8.716	6.442	16.35	17.67	11.84	6.202
95% confidence interval	3.292-3.513	3.223-3.464	3.423-3.697	3.236-3.452	7.897-8.799	5.745-6.401	4.839-5.372	3.513-3.766
	ΔperM::perM$_{mtb}$				*ΔperM::ftsB$_{mtb}$*			
**[Mg^{2+}] μM**	**25**	**100**	**250**	**2000**	**25**	**100**	**250**	**2000**
Sample size	185	184	202	232	260	194	275	255
Minimum	1.882	1.976	1.612	1.674	2.070	1.949	1.571	1.576
25th Percentile	3.111	2.872	2.735	2.525	3.923	2.960	2.976	2.749
Median	3.639	3.399	3.220	2.957	5.814	3.521	3.707	3.407
75th percentile	4.502	4.187	3.638	3.505	7.461	4.348	4.581	4.175
Maximum	8.407	6.690	8.336	6.846	16.01	8.876	9.055	7.770
95% confidence interval	3.690-3.996	3.432-3.701	3.192-3.430	3.001-3.213	5.796-6.457	3.555-3.860	3.749-4.039	3.420-3.682
