# Supplementary material for: Persistent Mycobacterium tuberculosis infection in mice requires PerM for successful cell division
Source: eLife. 2019 Nov 21;8:e49570. doi: 10.7554/eLife.49570 (PMC6872210; doi:10.7554/eLife.49570)
Supplement: Figure 6—source data 1. [file elife-49570-fig6-data1.pdf]

Figure 6 – Source data 1. Summary statistics of Figure 6C

	WT		*ΔperM*		*ΔperM::perM$_{mtb}$*		*ΔperM::ftsB$_{mtb}$*	
**(μm)**	**pH 7**	**pH 5.5**	**pH 7**	**pH 5.5**	**pH 7**	**pH 5.5**	**pH 7**	**pH 5.5**
Sample size	173	194	154	282	142	332	153	231
Minimum	1.616	1.665	1.888	1.969	1.748	1.605	1.830	1.799
25th Percentile	2.432	2.409	2.899	3.126	2.427	2.455	2.576	2.579
Median	2.829	2.806	3.413	4.061	2.773	2.825	3.152	2.957
75th percentile	3.195	3.315	4.475	5.642	3.383	3.394	3.660	3.629
Maximum	4.156	4.876	7.526	12.95	4.953	5.406	8.273	6.443
95% confidence interval	2.746-2.912	2.824-3.014	3.553-3.934	4.398-4.876	2.816-3.033	2.887-3.036	3.101-3.399	3.012-3.206
