# Supplementary material for: Persistent Mycobacterium tuberculosis infection in mice requires PerM for successful cell division
Source: eLife. 2019 Nov 21;8:e49570. doi: 10.7554/eLife.49570 (PMC6872210; doi:10.7554/eLife.49570)
Supplement: Figure 6—figure supplement 1—source data 1. [file elife-49570-fig6-figsupp1-data1.pdf]

Figure 6 – Source data 2. Summary statistics of Figure 6 – figure supplement 1B

	**WT**		***ΔperM***		***ΔperM::perM*$_{mtb}$**		***ΔperM::ftsB*$_{mtb}$**	
**(μm)**	**500μM**	**chelated**	**500μM**	**chelated**	**500μM**	**chelated**	**500μM**	**chelated**
Sample size	246	313	307	233	269	205	265	221
Minimum	1.664	1.317	1.614	2.091	1.746	1.653	1.762	1.490
25th Percentile	2.595	3.001	2.981	3.977	2.509	3.150	2.721	3.376
Median	3.066	3.576	3.557	4.899	3.046	3.854	3.212	4.017
75th percentile	3.513	4.135	4.288	5.884	3.547	4.507	3.724	4.682
Maximum	5.824	6.606	8.865	10.65	5.505	6.681	7.311	10.02
95% confidence interval	3.023-3.215	3.548-3.751	3.592-3.838	4.834-5.227	3.020-3.207	3.783-4.048	3.228-3.439	3.968-4.280
